# Overwintering of Vineyard Yeasts: Survival of Interacting Yeast Communities in Grapes Mummified on Vines

**DOI:** 10.3389/fmicb.2016.00212

**Published:** 2016-02-29

**Authors:** Matthias Sipiczki

**Affiliations:** Department of Genetics and Applied Microbiology, University of DebrecenDebrecen, Hungary

**Keywords:** yeasts, grape, Tokaj, molecular taxonomy, diversity, antagonism, wine

## Abstract

The conversion of grape must into wine involves the development and succession of yeast populations differing in species composition. The initial population is formed by vineyard strains which are washed into the must from the crushed grapes and then completed with yeasts coming from the cellar environment. As the origin and natural habitat of the vineyard yeasts are not fully understood, this study addresses the possibility, that grape yeasts can be preserved in berries left behind on vines at harvest until the spring of the next year. These berries become mummified during the winter on the vines. To investigate whether yeasts can survive in these overwintering grapes, mummified berries were collected in 16 localities in the Tokaj wine region (Hungary-Slovakia) in early March. The collected berries were rehydrated to recover viable yeasts by plating samples onto agar plates. For the detection of minority species which would not be detected by direct plating, an enrichment step repressing the propagation of alcohol-sensitive yeasts was also included in the process. The morphological, physiological, and molecular analysis identified 13 basidiomycetous and 23 ascomycetous species including fermentative yeasts of wine-making relevance among the 3879 isolates. The presence of viable strains of these species demonstrates that the grapes mummified on the vine can serve as a safe reservoir of yeasts, and may contribute to the maintenance of grape-colonizing yeast populations in the vineyard over years, parallel with other vectors and habitats. All basidiomycetous species were known phylloplane yeasts. Three Hanseniaspora species and pigmented *Metschnikowia* strains were the most frequent ascomycetes. Other fermentative yeasts of wine-making relevance were detected only in the enrichment cultures. *Saccharomyces* (*S. paradoxus, S. cerevisiae*, and *S. uvarum*) were recovered from 13% of the samples. No *Candida zemplinina* was found. The isolates with *Aureobasidium* morphology turned out to belong to *Aureobasidium subglaciale, Kabatiella microsticta*, or *Columnosphaeria fagi*. The ascomyceteous isolates grew at high concentrations of sugars with *Wickerhamomyces anomalus* being the most tolerant species. Complex interactions including antagonism (growth inhibition, contact inhibition, competition for nutrients) and synergism (crossfeeding) among the isolates and with *Botrytis cinerea* shape the composition of the overwintering communities.

## Introduction

Wine is the product of the activity of complex microbial communities in which fermentative yeasts and bacteria play the major roles. It has been demonstrated by numerous studies, that the vineyard flora is the primary source of inoculation of the grape must with yeasts (for a review, see Fleet et al., 2002). When the fermentation takes place in wineries regularly used for wine-making, the population of the grape yeasts is supplemented with residential winery yeasts. In spontaneous fermentation, these yeasts drive the fermentation process with successive dominance of less and more alcohol tolerant species. Although fermentative yeasts, including *Saccharomyces*, can occur on grapes (e.g., Combina et al., 2005; Schuller et al., 2005; Valero et al., 2007; Cordero-Bueso et al., 2011; Setati et al., 2012; Bokulich et al., 2014; Taylor et al., 2014), the winery-borne yeasts usually overgrow the grape-borne strains in advanced stages of fermentation performed in cellars and can dominate the process until its completion (e.g., Rosini, 1984; Martini, 1993; Vaughan-Martini and Martini, 1995; Egli et al., 1998; Gutierrez et al., 1999; Ciani et al., 2004; Santamarıa et al., 2005; Mercado et al., 2007; Di Maio et al., 2012).

The grape berries are colonized in the vineyard by both ascomyceteous and basidiomyceteous yeasts, and their communities change over time depending on the stage of ripening (for reviews, see Fleet et al., 2002; Bisson and Joseph, 2009; Barata et al., 2012). In a comprehensive study, Bourret et al. (2013) identified 53 yeast species on grapes in a Washington State vineyard. Other laboratories have found most of these species in many other wine-growing regions of the globe and found numerous additional species (e.g., Yanagida et al., 1992; Zahavi et al., 2002; Antunovics et al., 2003; Prakitchaiwattana et al., 2004; Raspor et al., 2006; Sipiczki, 2006; Renouf et al., 2007; Chavan et al., 2009; Brysch-Herzberg and Seidel, 2015; Nemcová et al., 2015; Setati et al., 2015).

The main factor determining the composition of the yeasts communities on the grape appears to be nutrient availability on the berry surface which increases with ripening. During the growth of the grape, before the unset of ripening, the surface yeast flora is dominated by basidiomyceteous genera (e.g., *Cryptococcus, Rhodosporidium, Rhodotorula, Sporobolomyces*) and the dimorphic ascomyceteous genus *Aureobasidium* (reviewed in Bisson and Joseph, 2009; Barata et al., 2012). These yeasts capable of growth in the nutrient-poor surface of the developing berries are also present on other parts of the grapevine and on the phylloplane of other plants (for a review, see Fonseca and Inacio, 2006). When the fruit begins to ripen, yeasts belonging to ascomyceteous genera (e.g., *Hanseniaspora, Metschnikowia, Candida*) start proliferating on the grape skin, probably due to nutrients leaking out through the thinning skin. Interestingly, *Saccharomyces*, the major wine yeast is not ubiquitous on the ripening grape and if present, only constitutes very small fractions of the yeast communities (Setati et al., 2012; Bokulich et al., 2014; Taylor et al., 2014). *Saccharomyces* strains were more frequently isolated from heavily damaged grapes (e.g., Mortimer and Polsinelli, 1999), where the juice of the grape became accessible to the yeasts through the skin lesions.

All yeasts present on the grape at harvest are washed into the must at crush. However, not all grape-borne yeasts are equally important for the process of turning the grape must into wine (vinification). The basidiomyceteous species are the least relevant group because they die off very quickly in the must due to their inability to ferment the juice sugars. The ascomyceteous yeast-like *Aureobasidium* does not survive in the wine either (Renouf et al., 2007). Among the fermentative species, *S. cerevisiae* and *S. uvarum* (*S. bayanus* var. *uvarum*) are the most important yeasts because they drive the alcoholic fermentation and release the most important metabolites into the fermenting wine. The non-*Saccharomyces* yeasts usually play secondary roles by fine-tuning the wine character or act as spoilage microorganisms producing off-flavors (for reviews, see Loureiro and Malfeito-Ferreira, 2003; Jolly et al., 2014).

Some important questions are still to be answered about how the fermentative non-phylloplane yeasts show up on the grapes. Insects attracted by damaged berries have been implicated in the dispersal of the yeasts, with honey bees (Goddard et al., 2010), wasps (Stefanini et al., 2012), and the fruit fly *Drosophila* assumed to act as vectors (Lam and Howell, 2015) and perhaps also to preserve the yeasts in their (hibernated or dehydrated) bodies over the winter until the next spring. It is pertinent to note here, that various yeasts species have been isolated from various *Drosophila* species collected in various habitats such as tree exudates, rotting cacti, rain forests, oak-pine forests, etc. (e.g., Dobzhansky et al., 1956; Phaff and Knapp, 1956; Starmer et al., 1976; Gilbert, 1980; Lachance et al., 1995; Morais et al., 2005). These yeasts can easily be vectored onto the ripe grape by the host insects. However, fermentative yeast species are detectable already in early stages of ripening when the berries are still sound (intact). In addition, an important fermentative yeast, *Saccharomyces* does not appear to be regularly associated with the *Drosophila* flies in the nature. In a recent study, *S. cerevisiae* was not detected in 296 flies captured in vineyards, grape waste (marc) piles and wineries of two Australian wine-growing regions during grape harvest (Lam and Howell, 2015). Moreover, it is not the fruit volatiles but the yeasts, that attract *Drosophila* flies (Becher et al., 2012). *Hanseniaspora* has been found to produce aromas that are attractive to *D. melanogaster* (Palanca et al., 2013). So, at least certain berries have to be colonized by yeasts before the flies come, otherwise they would not come.

Vineyard soil is a potential source of the grape yeasts because the berries which fall to the ground during ripening and at harvest harbor large populations of yeasts. The work of Cordero-Bueso et al. (2011) describing different yeast populations in musts produced from grapes of wineyards in which different soil management methods were used indicates, that the condition of the soil can have an impact on the yeast communities of the grape. However, the soil is a rather unfavorable habitat for yeast overwintering because the soil microorganisms decompose the organic materials (for a review, see Treseder and Lennon, 2015) including the berries and their yeast colonists. Parle and Di Menna (1966) found very few fermentative yeasts in summer samples of vineyard soil and only *Kloeckera (Hanseniaspora) apiculata* in winter samples.

The goal of this study was to investigate an alternative possibility, the survival of grape yeasts in berries left-behind on the vine at harvest. These berries turn dry during winter and become mummified. To investigate whether the yeasts colonizing the ripe grape in autumn can survive the winter in these berries, mummified grapes were collected in the Tokaj wine-growing region in March. The peculiarity of this region is the extensive botrytisation leading to noble rotting of the grape on the vine. Noble rot is a benevolent *Botrytis*-generated process associated with dehydration (drastic increase of sugar content) and intense colonization of the ruptured berries by complex microbial consortia (Antunovics et al., 2003; Magyar and Bene, 2004). The collected grape mummies were rehydrated and used for recovering viable yeasts. The taxonomic examination of the recovered yeasts identified high numbers of basidiomyceteous and ascomyceteous yeast species, demonstrating, that the grape mummified on the vine also may contribute to the maintenance of the continuity of the vineyard yeast microflora over consecutive years.

## Materials and methods

### Sample collection and yeast isolation

**Figure 1:**
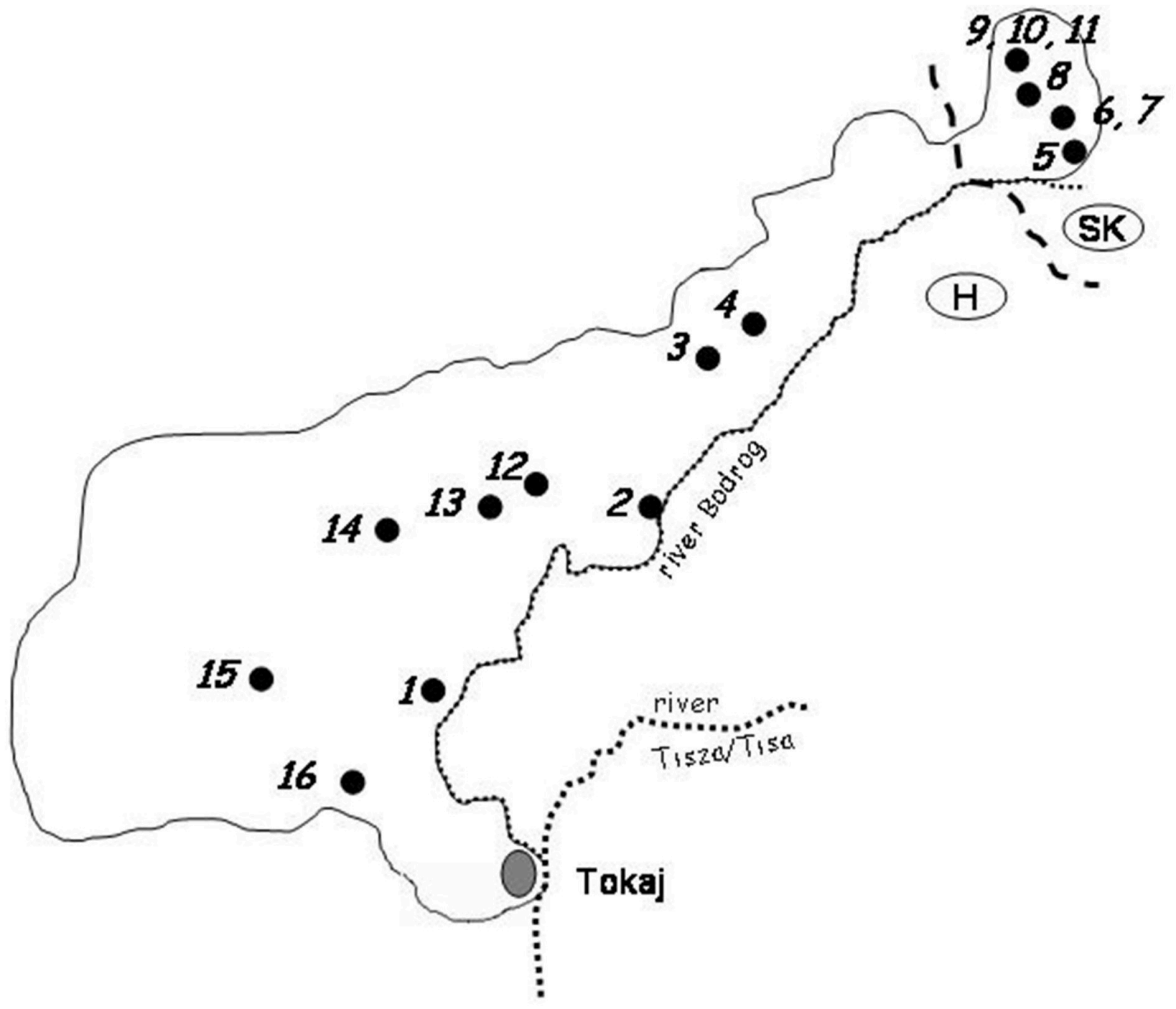
**Geographic locations of vineyards in which samples were collected**. 1. Szegi; 2. Sárazsadány; 3. Hercegkút; 4. Hercegkút; 5. Viničky; 6. Bara; 7. Bara; 8. Černochov; 9. Malá Tŕňa; 10. Malá Tŕňa; 11. Malá Tŕňa; 12. Tolcsva; 13. Tolcsva; 14. Erdöbénye; 15. Abaujszántó; 16. Mád.

Three bunches of mummified grapes were collected from vines in each of the16 vineyards selected for the investigation (Figure 1). Five berries were picked aseptically from each bunch and placed in a sterile test-tube. As the berries were completely desiccated, 2.5 ml of YEL (1% yeast extract, 2% glucose) was added to the test-tube to rehydrate them. After 1 h of incubation at room temperature, the soaked berries were macerated with a sterile spatula and homogenized with intense vortexing. 10-μl aliquots of the homogenized sample were spread onto YEA (YEL supplemented with 2% agar) plates. The rest of the sample was incubated at room temperature overnight. Then 10-μl aliquots were plated on YEA again and 0.5-ml volumes were transferred into test-tubes containing 4.5 ml of enrichment medium (0.68% yeast nitrogen base, 1.1% raffinose, and 9% ethanol). This medium selectively supports the growth of the yeasts which tolerate high ethanol concentrations and utilize raffinose as a carbon source (Sampaio and Goncalves, 2008). The tube was incubated at 10°C. After 4 weeks of incubation, 10-μl aliquots of the enrichment culture were plated on YEA (when the cell number was high, the aliquots were diluted before plating). After 7 days of incubation at room temperature, colonies (max 150) were randomly isolated from the plates for each grape sample in order to obtain representative collections of pure isolates. The isolates were stored at 5°C on YEA plates and reinoculated onto fresh plate every month.

### Phenotypic characterization of isolates

Colony morphology (color, surface ornamentation, production of pigmented zone in the medium) on YEA plates was examined and recorded for each isolate. All isolates were tested for the ability to assimilate 14 compounds as carbon-sources and lysine as a nitrogen source by replica-plating of 5-day old YEA cultures onto assimilation test plates (0.68% DIFCO yeast nitrogen base and 2% agar) supplemented with the carbon sources and onto SMA-lysine plates (2% glucose, 2% agar, 0.5% lysine and vitamins). The carbon sources tested were: cellobiose, ethanol, galactose, glucose, inulin, maltose, melesitose, melibiose, raffinose, rhamnose, ribose, saccharose, trehalose, and xylose. Growth was evaluated after 7 days of incubation at room temperature.

### Molecular taxonomy

For taxonomic identification of the isolates, the D1/D2 domains of their large subunit ribosomal RNA genes and the ITS regions were amplified and sequenced with the primer pairs NL1-NL2 and ITS1-ITS4 as described earlier (Sipiczki, 2003). To assess their taxonomic positions, the resultant sequences were used to identify similar sequences in the GenBank database with the MEGABLAST-querying service of NCBI (http://blast.ncbi.nlm.nih.gov/Blast.cgi). As the GenBank entries are not checked for the correctness of their taxonomic assignment by the depositors the D1/D2 sequences of the isolates were then aligned with the D1/D2 sequences of the type strains of the species whose sequences were found highly similar in the GenBank search. For this, the sequences of the type strains were downloaded from the CBS database (http://www.cbs.knaw.nl/Collections/). The exact sequence similarity with the type strain sequences (number of identical nucleotides) was determined by pairwise Blast alignment using the bl2seq algorithm available at NCBI.

### Osmotolerance

Dense suspensions of cells (~10^7^ cells ml^−1^) were prepared from early stationary phase cultures of the isolates cultivated in YEL at room temperature for 2 days. 5-μl aliquots of the suspensions were dropped on YEA plates supplemented with 2, 20, 30, 40, and 50% of a 1:1 mixture of glucose and fructose. Yeast growth was evaluated after 5 days of incubation at 25°C by comparing the thickness of the spots.

### Interaction tests

**Figure 2:**
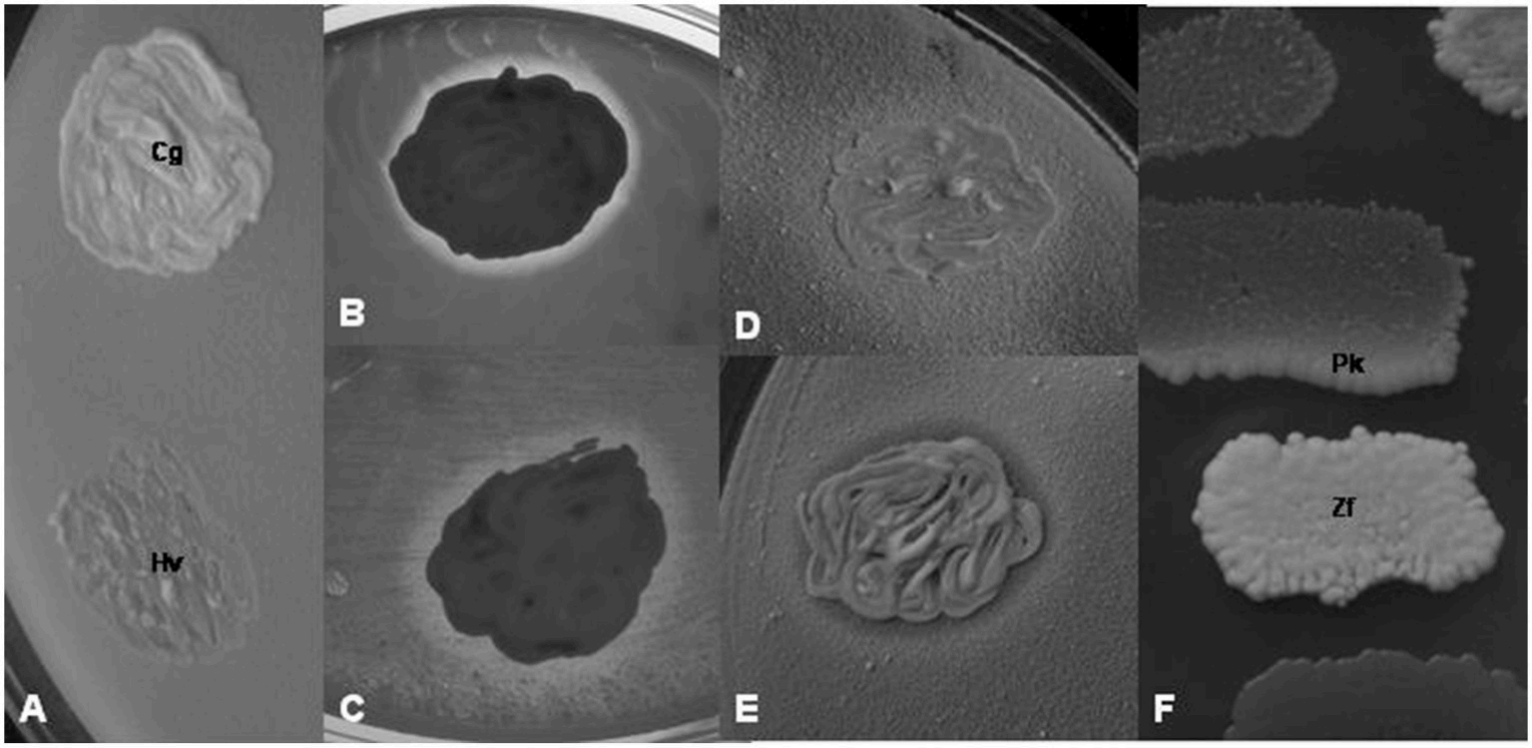
**Yeast-yeast interactions. (A)** *L. thermotolerans* 8/2z-3 (lawn) shows no interaction with *Ca. glabrata* 14/1z-1 (Cg) but inhibits the growth of *H. vineae* 11/1z-4 (Hv). **(B)** Clear inhibition zone in the *Ca. glabrata* 14/1z-1 lawn around the *W. anomalus* 15/z-7 colony. **(C)** Turbid inhibition zone in the *H. vineae* 11/1z-4 lawn around the *W. anomalus* 15/z-7 colony. **(D)** Synergistic effect of the *P. scaptomyzae* 12/z-4 colony on the *T. delbrueckii* 1/1/z-10 lawn. **(E)** Dual effect: inhibition zone and crossfeeding of the *Kl. dobzhanskii* 9/z-6 lawn by *the P. scaptomyzae* 12z-4 colony. **(F)** Crossfeeding of the melibiose-minus *P. kluyveri* 11/2-104 colony (Pk) by the melibiose-positive *Zt. florentina* 7z-11 colony (Zf) on the medium containing melibiose as carbon source. **(B)** and **(C)** were photographed with transmitted light.

(a) Interactions among yeasts. For testing the yeast isolates for interactions with other yeast isolates, 5-day old cultures grown on YEA plates were used. Dense suspensions (~10^8^ cells ml^−1^) were prepared from each isolate in 2 ml of sterile water, and YEA plates were flooded with the suspensions to obtain homogeneous lawns of cells. The rests of the suspensions were pored off. After drying the surface of the plates, loopful amounts of other isolates were smeared on the plates to form spots of ~5 mm in diameter (Figure 2). The plates were then incubated at 25°C for 7 days and examined at regular time intervals for the growth intensity of the spots and the lawn around the spots.

(b) Interactions between yeast isolates and *B. cinerea*. The effect of the yeast isolates on fungal growth was examined on YEA and PDA (Potato Dextrose Agar, Scharlab S.L.) plates flooded with suspensions of *Botrytis* conidia. The suspension of conidia was obtained by washing the surface of 2-week old *B. cinerea* 980 (Sipiczki, 2006) cultures grown on PDA at room temperature with sterile water. After removing the rest of the suspension and drying the surface of the plates, yeast isolates were inoculated onto the lawn of conidia as described above. The plates were incubated at 20°C for 2 weeks and examined at regular time intervals.

## Results

### Yeast isolation, characterization, and taxonomic identification

Yeast colonies were obtained with both isolation methods (with and without enrichment), even from those enrichment cultures which did not become turbid (no yeast growth). 3879 isolated colonies were examined for morphology, tested for the utilization of 15 compounds as sole carbon or nitrogen sources and then clustered into groups on the basis of their phenotypes. From representatives of the phenotypic groups, D1/D2 domain regions of the rDNA arrays were amplified and sequenced. Based on the similarity of the sequences to those of the type strains of known species, all but one group could be assigned to known species. 13 basidiomyceteous and 23 ascomyceteous species were identified in this way among the isolates.

Three groups of basidiomyceteous isolates could not be assigned to single species because their D1/D2 sequences were equally similar to the D1/D2 sequences of more than one type strain. In one of these groups, the similarity search found three identical type-strain sequences: *Cryptococcus magnus, Filobasidium elegans*, and *Filobasidium floriforme*. These species are indistinguishable when their D1/D2 sequences are compared but can be separated when certain physiological traits are also examined (Fell et al., 2000; Fonseca et al., 2011; Kwon-Chung, 2011). The assimilation tests of the isolates assigned this group to *Cr. magnus* because they grew on galactose (difference from *F. elegans*) and inulin (difference from both *F. elegans* and *F. florioformae*). A different group of isolates differed by one nucleotide from both *Sporobolomyces coprosmae* and *Sp. oryzicola*, a pair of very closely related sibling species which cannot be distinguished by D1/D2 sequencing (Scorzetti et al., 2002). Unfortunately, they cannot be differentiated by their growth reactions on the carbon compounds commonly tested in taxonomical studies, either (Hamamoto et al., 2011). Therefore, the ITS region also was sequenced from one of the isolates. Its sequence differed by 2 substitutions from *Sp. coprosmae* and 3 substitutions from *Sp. oryzicola*. Additional genes should be sequenced to reinforce the somewhat closer relationship to the former species. The third group showed 100% identity with the *Curvibasidium pallidicorallinum* type strain and differed from the *Rhodotorula nothofagi type* strain only by one substitution. The latter difference was due to an unambiguous nucleotide in the type strain sequence, so the somewhat higher similarity to *Cu. pallidicorallinum* was not relevant. The sugar assimilation tests indicated conspecificity with *Cu. pallidicorallinum:* the isolates grew on maltose, trehalose, and inulin, which are not assimilated by *R. notophagi* (Sampaio, 2011a,b).

The group represented by the isolate 6–13 in Table 1 could not be assigned to any species because its D1/D2 domain was very different from all type-strain sequences. Its closest relative type strain was *Cr. keelungensis* CBS 10876^T^, from which it differed by 24 substitutions (4%). Interestingly, it showed 99 to 100% identity to D1/D2 sequences of taxonomically uncharacterized yeast strains isolated from tree leaves, floral nectar (Alvarez-Perez and Herrera, 2013), and Iberian Pyrite Belt (Gadanho et al., 2006), indicating conspecificity with a hitherto non-described species which seems to live in diverse habitats.

**Table 1:** **D1/D2 sequence differences of selected representatives of the phenotypic groups of isolates from type strains of the most similar species**.

**Isolate**			**Most similar type/reference strain**		**Sequence difference (number of substitutions/indels)**
**Identification number**	**Location of sample collection**	**D1/D2 accession number**	**Taxonomic name**	**D1/D2 accession number**	
**ASCOMYCOTA, PEZIZOMYCOTINA**					
10–59	Malá Tŕňa	KU254559	*Aureobasidium subglaciale* CBS 123387^T^	FJ150913	0
1/2–11	Szegi		*Kabatiella microsticta* CBS 342.66 *Columnosphaeria fagi* CBS 171.93	FJ150952 AY016359	0 0
6/1–5	Bara	KU254558	*Kabatiella microsticta* CBS 342.66 *Columnosphaeria fagi* CBS 171.93	FJ150952 AY016359	0 0
13/1–4	Tolcsva		*Kabatiella microsticta* CBS 342.66 *Columnosphaeria fagi* CBS 171.93	FJ150952 AY016359	0 0
**ASCOMYCOTA, SACCHAROMYCOTINA**					
14/1z-1	Erdöbénye	KT933331	*Candida glabrata* CBS 138^T^	AF399771	1
11/1–54	Malá Tŕňa		*Candida oleophila* CBS 2219^T^	U45793	0
11/1-55	Malá Tŕňa	KT122406	*Candida oleophila* CBS 2219^T^	U45793	0
7-9	Bara		*Kregervanrija fluxuum* CBS 639^T^	U70247	3^z^
1/1z-2	Szegi		*Hanseniaspora osmophila* CBS 313^T^	U84228	0
2z-22	Sárazsadány		*Hanseniaspora osmophila* CBS 313^T^	U84228	0
4–3	Hercegkút		*Hanseniaspora osmophila* CBS 313^T^	U84228	0
4z-5	Hercegkút		*Hanseniaspora osmophila* CBS 313^T^	U84228	0
5/1z-3	Vinièky		*Hanseniaspora osmophila* CBS 313^T^	U84228	0
5/2–6	Viničky		*Hanseniaspora osmophila* CBS 313^T^	U84228	1^z^
5/2z-6	Viničky		*Hanseniaspora osmophila* CBS 313^T^	U84228	0
5/2z-11	Viničky		*Hanseniaspora osmophila* CBS 313^T^	U84228	1^z^
7/2z-1	Bara		*Hanseniaspora osmophila* CBS 313^T^	U84228	0
8–3	Černochov	KT933332	*Hanseniaspora osmophila* CBS 313^T^	U84228	0
8z-4	Černochov	KT175536	*Hanseniaspora osmophila* CBS 313^T^	U84228	0
9z-3	Malá Tŕňa		*Hanseniaspora osmophila* CBS 313^T^	U84228	0
11/2z-2	Malá Tŕňa		*Hanseniaspora osmophila* CBS 313^T^	U84228	0
12/2z-3	Tolcsva		*Hanseniaspora osmophila* CBS 313^T^	U84228	0
13/2–90	Tolcsva		*Hanseniaspora osmophila* CBS 313^T^	U84228	0
13/2z-5	Tolcsva		*Hanseniaspora osmophila* CBS 313^T^	U84228	0
15z-4	Abaújszántó		*Hanseniaspora osmophila* CBS 313^T^	U84228	0
1–3	Szegi		*Hanseniaspora uvarum* CBS 314^T^	U84229	0
1z-2	Szegi		*Hanseniaspora uvarum* CBS 314^T^	U84229	0
1/1–32	Szegi		*Hanseniaspora uvarum* CBS 314^T^	U84229	0
2–4	Sárazsadány		*Hanseniaspora uvarum* CBS 314^T^	U84229	0
4/1–6	Hercegkút		*Hanseniaspora uvarum* CBS 314^T^	U84229	0
7–3	Bara		*Hanseniaspora uvarum* CBS 314^T^	U84229	0
7/2–17	Bara		*Hanseniaspora uvarum* CBS 314^T^	U84229	0
8/2-28	Černochov		*Hanseniaspora uvarum* CBS 314^T^	U84229	0
9/1–3	Malá Tŕňa		*Hanseniaspora uvarum* CBS 314^T^	U84229	0
9/1–66	Malá Tŕňa	KT156710	*Hanseniaspora uvarum* CBS 314^T^	U84229	0
11/1–10	Malá Tŕňa		*Hanseniaspora uvarum* CBS 314^T^	U84229	0
13/2–4	Tolcsva		*Hanseniaspora uvarum* CBS 314^T^	U84229	0
14/1–7	Erdöbénye		*Hanseniaspora uvarum* CBS 314^T^	U84229	0
15/1–10	Abaújszántó		*Hanseniaspora uvarum* CBS 314^T^	U84229	0
15/2–1	Abaújszántó		*Hanseniaspora uvarum* CBS 314^T^	U84229	0
16–5	Mád		*Hanseniaspora uvarum* CBS 314^T^	U84229	0
16/2–2	Mád		*Hanseniaspora uvarum* CBS 314^T^	U84229	0
1/1–5	Szegi		*Hanseniaspora vineae* CBS 2171^T^	U84224^x^	3
11/1z-4	Malá Tŕňa	KT933333	*Hanseniaspora vineae* CBS 2171^T^	U84224^x^	1
12/1z-2	Tolcsva		*Hanseniaspora vineae* CBS 2171^T^	U84224^x^	4
12/1z-5	Tolcsva		*Hanseniaspora vineae* CBS 2171^T^	U84224^x^	4
5z-9	Viničky		*Kluyveromyces dobzhanskii* CBS 2104^T^	U69575	0
5z-17	Viničky		*Kluyveromyces dobzhanskii* CBS 2104^T^	U69575	0
7/1z-5	Bara		*Kluyveromyces dobzhanskii* CBS 2104^T^	U69575	0
9/z-1	Malá Tàòa		*Kluyveromyces dobzhanskii* CBS 2104^T^	U69575	0
9z-6	Malá Tŕňa	KT122408	*Kluyveromyces dobzhanskii* CBS 2104^T^	U69575	0
10z-4	Malá Tŕňa		*Kluyveromyces dobzhanskii* CBS 2104^T^	U69575	0
10/1z-1	Malá Tŕňa		*Kluyveromyces dobzhanskii* CBS 2104^T^	U69575	0
2/2z-8	Sárazsadány		*Lachancea thermotolerans* CBS 6340^T^	U69581	0
5/1z-7	Viničky		*Lachancea thermotolerans* CBS 6340^T^	U69581	0
8z-1	Černochov	KT175534	*Lachancea thermotolerans* CBS 6340^T^	U69581	0
8/2z-3	Černochov	KT933334	*Lachancea thermotolerans* CBS 6340^T^	U69581	0
9/1-15	Malá Tŕňa		*Lachancea thermotolerans* CBS 6340^T^	U69581	0
9/1z-4	Malá Tŕňa		*Lachancea thermotolerans* CBS 6340^T^	U69581	0
10/2z-4	Malá Tŕňa		*Lachancea thermotolerans* CBS 6340^T^	U69581	0
11–27	Malá Tŕňa		*Lachancea thermotolerans* CBS 6340^T^	U69581	0
11z-1	Malá Tŕňa		*Lachancea thermotolerans* CBS 6340^T^	U69581	0
11/2–112	Malá Tŕňa		*Lachancea thermotolerans* CBS 6340^T^	U69581	0
14/1z-2	Erdöbénye		*Lachancea thermotolerans* CBS 6340^T^	U69581	0
3z-1	Hercegkút		*Metschnikowia* sp. 11-1090 clone d4	KM350710	11
5z-6	Viničky	KT933337	*Pichia fermentans* CBS 187^T^	U75726	2
5z-10	Viničky		*Pichia fermentans* CBS 187^T^	U75726	2^*y*^
5z-12	Viničky		*Pichia fermentans* CBS 187^T^	U75726	1
11/2–104	Malá Tŕňa	KT156709	*Pichia kluyveri* CBS 188^T^	U75727^x^	1
4/1–34	Hercegkút	KT933335	*Pichia membranifaciens* CBS 2763^T^	DQ198963	3
15/1z-9	Abaújszántó		*Pichia membranifaciens* CBS 2763^T^	DQ198963	3
12z-4	Tolcsva	KT933336	*Pichia scaptomyzae* CBS 8167^T^	AB045136	0
12z-14	Tolcsva		*Pichia scaptomyzae* CBS 8167^T^	AB045136	0
3/1z-5	Hercegkút		*Saccharomyces cerevisiae* CBS 1171^NT^	U44806	0
14/z-1	Erdöbénye	KT933338	*Saccharomyces cerevisiae* CBS 1171^NT^	U44806	0
3z-28	Hercegkút		*Saccharomyces paradoxus* CBS 432^NT^	U68555	2
3/2z-5	Hercegkút		*Saccharomyces paradoxus* CBS 432^NT^	U68555	2
5z-7	Viničky		*Saccharomyces paradoxus* CBS 432^NT^	U68555	2
7/2z-2	Bara		*Saccharomyces paradoxus* CBS 432^NT^	U68555	2
7/2z-3	Bara		*Saccharomyces paradoxus* CBS 432^NT^	U68555	2
10z-2	Malá Tŕňa	KT122407	*Saccharomyces paradoxus* CBS 432^NT^	U68555	2
4/2z-11	Hercegkút	KT933339	*Saccharomyces uvarum* CBS 395^T^	AJ279065	0
1/1z-1	Szegi		*Torulaspora delbrueckii* CBS 1146^T^	U72156	0
1/1z-10	Szegi	KT933340	*Torulaspora delbrueckii* CBS 1146^T^	U72156	0
15z-7	Abaújszántó	KT933341	*Wickerhamomyces anomalus* CBS 5759^T^	U74592	0
8z-2	Černochov	KT175535	*Zygoascus meyerae* CBS 7521^T^	AY447014	0
4–24	Hercegkút	KT933342	*Zygosaccharomyces bailii* CBS 680^T^	U72161	0
8–29	Černochov		*Zygosaccharomyces bailii* CBS 680^T^	U72161	0
2z-30	Sárazsadány		*Zygotorulaspora florentina* CBS 746^T^	U72165	0
3/2–1	Hercegkút		*Zygotorulaspora florentina* CBS 746^T^	U72165	0
7z-11	Bara	KU254556	*Zygotorulaspora florentina* CBS 746^T^	U72165	0
9/2z-9	Malá Tŕňa		*Zygotorulaspora florentina* CBS 746^T^	U72165	0
**BASIDIOMYCOTA, AGARIMYCOTINA**					
13/1–34	Tolcsva	KT933343	*Bulleromyces albus* CBS 500^T^	AF416643	0
6/2–10	Bara	KT933344	*Cryptococcus carnescens* CBS 973^T^	AB035054	0
7/2–10	Bara		*Cryptococcus flavescens* CBS 942^T^	AB035042	0
10/2–3	Malá Tŕňa		*Cryptococcus flavescens* CBS 942^T^	AB035042	0
8–30	Černochov	KT933345	*Cryptococcus flavescens* CBS 942^T^	AB035042	0
12/2–18	Tolcsva		*Cryptococcus flavescens* CBS 942^T^	AB035042	0
13/1–29	Tolcsva		*Cryptococcus flavescens* CBS 942^T^	AB035042	0
6–13	Bara	KT001494	*Cryptococcus keelungensis* CBS 10876^T^	EF621562	24
6–21	Bara	KT933346	*Cryptococcus magnus* CBS 140^T^ *Filobasidium elegans* CBS 7640 *Filobasidium floriforme* CBS 6241	AF181851 AF181548 AF075498	0 0 0
7/1–40	Bara	KT933352	*Cryptococcus magnus* CBS 140^T^ *Filobasidium elegans* CBS 7640 *Filobasidium floriforme* CBS 6241	AF181851 AF181548 AF075498	0 0 0
7/1–55	Bara		*Cryptococcus magnus* CBS 140^T^ *Filobasidium elegans* CBS 7640 *Filobasidium floriforme* CBS 6241	AF181851 AF181548 AF075498	1 1 1
10/2–10	Malá Tŕňa	KT933347	*Cryptococcus stepposus* CBS 10265^T^	DQ222456	0
11/2–10	Malá Tŕňa	KT933348	*Cryptococcus victoriae* CBS 8685^T^	AF363647	2
12/2–50	Tolcsva		*Cryptococcus victoriae* CBS 8685^T^	AF363647	2
13/2–49	Tolcsva	KU254557	*Cryptococcus carnescens* CBS 973^T^ *Cryptococcus victoriae* CBS 8685^T^	AB035054 AF363647	8 8
13/1–37	Tolcsva	KT933349	*Cryptococcus wieringae* CBS 1937^T^	AF181541	0
7/1–56	Bara	KT933353	*Holtermanniella festucosa* VKM Y-2930^T^	AY462119	6
**BASIDIOMYCOTA, PUCCINIOMYCOTINA**					
8/1–14	Černochov	KT933350	*Curvibasidium cygneicollum* CBS 4551^T^	AF189928	0
10–10	Malá Tŕňa		*Curvibasidium cygneicollum* CBS 4551^T^	AF189928	0
10–20	Malá Tŕňa		*Curvibasidium cygneicollum* CBS 4551^T^	AF189928	0
15–23	Abaújszántó		*Curvibasidium cygneicollum* CBS 4551^T^	AF189928	0
15–25	Abaújszántó		*Curvibasidium cygneicollum* CBS 4551^T^	AF189928	1
6–23	Bara		*Curvibasidium pallidicorallinum* CBS 9091^T^ *Rhodotorula nothofagi* CBS 8166^T^	AF444736 AF189950^w^	0 1
7/1–1	Bara	KT156708	*Curvibasidium pallidicorallinum* CBS 9091^T^ *Rhodotorula nothofagi* CBS 8166^T^	AF444736 AF189950^w^	0 1
7/1–2	Bara		*Curvibasidium pallidicorallinum* CBS 9091^T^ *Rhodotorula nothofagi* CBS 8166^T^	AF444736 AF189950^w^	0 1
7/1–50	Bara		*Curvibasidium pallidicorallinum* CBS 9091^T^ *Rhodotorula nothofagi* CBS 8166^T^	AF444736 AF189950^w^	0 1
9–25	Malá Tŕňa	KT933351	*Curvibasidium pallidicorallinum* CBS 9091^T^ *Rhodotorula nothofagi* CBS 8166^T^	AF444736 AF189950^w^	1 2
9–50	Malá Tŕňa		*Curvibasidium pallidicorallinum* CBS 9091^T^ *Rhodotorula nothofagi* CBS 8166^T^	AF444736 AF189950^w^	0 1
11–2	Malá Tŕňa		*Curvibasidium pallidicorallinum* CBS 9091^T^ *Rhodotorula nothofagi* CBS 8166^T^	AF444736 AF189950^w^	0 1
13/1z-1	Tolcsva		*Curvibasidium pallidicorallinum* CBS 9091^T^ *Rhodotorula nothofagi* CBS 8166^T^	AF444736 AF189950^w^	0 1
13/1–16	Tolcsva	KT933354	*Rhodotorula graminis* CBS 2826^T^	AF070431	0
6/2–4	Bara	KT933355	*Sporobolomyces coprosmae* CBS 7899^T^ *Sporobolomyces oryzicola* CBS 7228^T^	AF189980 AF189990	1 1

T , Type strain;

NT , neotype strain;

x , contains one ambiguous nucleotide: N (A, G, C or T);

Y , isolate contains two ambiguous nucleotides: S (G or C) and Y (C or T);

z , isolate contains one ambiguous nucleotide: Y (C or T);

w , contains one ambiguous nucleotide: Y (C or T);

*^z^, isolate contains two ambiguous nucleotides: Y (C or T) and R (A or G)*.

Almost all samples harbored cells, that produced colonies showing the morphology characteristic of the ascomycetous yeast-like fungus *Aureobasidium pullulans*. These rapidly growing colonies contained yeast-like cells and septate hyphae producing lateral blastospores. Since *A. pullulans* plays a very marginal role in wine-making and is quite ubiquitous on the phylloplane, only a few isolates of this morphology were characterized taxonomically in this study. Unexpectedly, their D1/D2 sequences showed closer relationship to *A. glaciale, Kabatiella microsticta*, and *Columnosphaeria fagi* than to *A. pullulans*. The examples shown in Table 1 have D1/D2 sequences identical with those of these species and differ from that of the *A. pullulans* type strain by 17 and 4 substitutions, respectively.

The isolates producing pigmented halos in the medium around their colonies appeared to belong to the *pulcherrima* clade of *Metschnikowia* (Lachance, 2011). However, their exact taxonomic position could not be determined because their D1/D2 sequences were diverse and different from the corresponding sequences of all type strains of the clade. For example, the GenBank database sequences most similar to the example shown in Table 1 were KM350710 and KM275362 which were cloned from the rDNA arrays of the *M. sinensis* and *M. andauensis* type strains CBS 10359^T^ and CBS 10357^T^, respectively.

Tables S1, S2 show the occurrence and relative abundance of species in the 48 samples. The most frequently encountered species belonged to the genera *Metschnikowia, Hanseniaspora, Cryptococcus* before enrichment and *Kluyveromyces, Lachancea*, and *Pichia* after enrichment. Basidiomyceteous yeasts were detected in 69% of the samples before enrichment and only in 4% of the samples after enrichment (Table S2), indicating that the enrichment conditions were lethal to this group. The yeasts most frequently occurring in the enrichment cultures were strains of the ascomycetous genera *Metschnikowia* (in 33%), *Lachancea thermotolerance* (in 31%), and *Hanseniaspora osmophila* (in 31%; Table S1). Among these species only *L. thermotolerance* can utilize raffinose as carbon source. The other two species must have gained energy from alternative sources released from the dehydrated berry tissues and could overgrow the other yeasts due to their better ability to tolerate the high alcohol concentration and the low incubation temperature. *Saccharomyces* strains were found only in enriched cultures and only in 6 vineyards: *S. paradoxus* in 4*, S. cerevisiae* in 2*, S. uvarum* in 1 vineyard.

Lysine utilization was included in the phenotypic characterization of the isolates because one of the most frequently used methods in wine microbiology to identify *Saccharomyces* versus non-*Saccharomyces* yeasts is plating on lysine agar. *Saccharomyces* does not grow on lysine as a sole nitrogen source, therefore only non-*Saccharomyces* yeasts will grow on these plates (Angelo and Siebert, 1987). Consistent with this, none of the three *Saccharomyces* species identified in this study could utilize lysine as a nitrogen source. However, certain *Hanseniaspora, Candida glabrata, Zygoascus meyerae, Zygosaccharomyces bailii, Kl. Dobzhanskii*, and *L. thermotolerans* isolates identified by sequencing were also lysine minus.

### Osmotolerance

The results are shown in Table 2. Surprisingly, only 7 isolates grew at 50% sugar, but neither the *Saccharomyces* species nor *Zs. bailii* were among them. The most osmotolerant species was *Wickerhamomyces anomalus*.

**Table 2:** **Osmotolerance of representative isolates of ascomyceteous yeast species**.

**Species**	**Isolate**	**Growth on media supplemented with 1:1 fructose:glucose**			
		**2%**	**30%**	**40%**	**50%**
*Candida glabrata*	14/1z-1	+++	+++	++	+
*Candida oleophila*	11/1–55	+++	+++	++	+
*Hanseniaspora osmophila*	8-3	+++	+++	++	+
*Hanseniaspora uvarum*	9/1-66	+++	+++	+	(+)
*Hanseniaspora vineae*	11/1z-4	+++	+++	+(+)	(+)
*Kluyveromyces dobzhanskii*	9z-6	+++	+++	++(+)	–
*Lachancea thermotolerans*	8/2z-3	+++	+++	++(+)	+
*Metschnikowia sp*.	11/1-3	+++	+++	++	+
*Pichia fermentans*	5z-6	+++	+++	–	–
*Pichia kluyveri*	11/2–104	+++	+++	+	–
*Pichia membranifaciens*	4/1-34	+++	+++	++(+)	–
*Pichia scaptomyzae*	12z-4	+++	+++	+	–
*Saccharomyces cerevisiae*	14z-1	+++	+++	++	–
*Saccharomyces paradoxus*	10z-2	+++	+++	++	–
*Saccharomyces uvarum*	4/2z-11	+++	+++	++	–
*Torulaspora delbrueckei*	1/1z-10	+++	+++	++	+
*Wickerhamomyces anomalus*	15z-7	+++	+++	++(+)	++
*Zygoascus meyerae*	8z-2	+++	+++	+	–
*Zygosaccharomyces bailii*	4–24	+++	+++	++	–
*Zygotorulaspora florentina*	7z-11	+++	+++	++	–

*+, growth; (+), weak growth; –, no growth*.

### *Antagonistic* and *Synergistic* interactions among *Yeast* isolates

Negative interaction (growth inhibition) was detected as an inhibition zone in the sensitive lawn around the colony of the antagonist (Figures 2B,C) or as the inhibition of the growth of the sensitive colony on the lawn of the antagonistic isolate (Figure 2A). Two types of inhibition zones could be distinguished: clear (Figure 2B) and turbid (Figure 2C) zones. The positive, growth intensifying effect was noticed as a halo of stronger growth of the lawn (lawn thickening) around the colony of the isolate which had such effect (Figure 2D). In a few combinations of isolates simultaneous antagonistic and synergistic effects could be seen (Figure 2E). The results of the interaction tests carried out with isolates representing all ascomycetous yeasts are shown in Table 3.

**Table 3:** **Yeast antagonism**.

**Lawn**		**Inoculated on the lawn**																			
**Species**	**Isolate**	**14/1z-1**	**11/1–55**	**8-3**	**9/1–66**	**11/1z-4**	**9z-6**	**8/2z-3**	**11/1–3**	**4/1–34**	**12z-4**	**5z-6**	**11/2–104**	**10z-2**	**14z-1**	**4/2z-11**	**1/1z-10**	**15z-7**	**8z-2**	**4–24**	**7z-11**
*Candida glabrata*	14/1z-1	–	–	–^*^	–^*^	–^*^	1	1	0.5	–	–	–	–	–	–	–	–	2	–	–	–
*Candida oleophila*	11/1–55	–	–	–	–	–^*^	0.5	–	–^*^	–	–	–	–	–	–	–	–	1^*^	–	–	–
*Hanseniaspora osmophila*	8–3	±	2^T^	–	–	±	–	–^*^	–^*^	±	–^*^+	–^*^	–^*^	–^*^	–^*^	–^*^	±	–^*^+	–	–^*^	–^*^
*Hanseniaspora uvarum*	9/1–66	–	–	+^*^	–	–^*^	–	–^*^	–^*^	–^*^	–^*^	–^*^	–^*^	–^*^	–^*^	–^*^	–^*^	–^*^	–	–^*^	–^*^
*Hanseniaspora vineae*	11/1z-4	–	–	–^*^	–^*^	–	–	–^*^	–^*^	–^*^^+^	–^*^^+^	–^*^	–^*^	–^*^	–^*^	–^*^	1^t^^*^	3^t^^*^	–	–^*^	–^*^
*Kluyveromyces dobzhanskii*	9z-6	–	–	–	–	–	–	–	2^t^^*^	2^T^	1^*t*^+	–	0.5	1	1^T^	0.5	–	1^*^	–	0.5	1.5
*Lachancea thermotolerans*	8/2z-3	–	–	–^*^	–^*^	–^*^	1^T^	–	–^*^	1^*t*^+	±	–^*^	–^*^	–^*^	–	–	–	0.5^*^	–	–	–
*Metschnikowia sp*.	11/1–3	–	–	–	–	–	2^T^	1^T^	–	1^T^	–	–	–	0.5	–	0.5	1^T^	1^T^	0.5^T^	–	–
*Pichia fermentans*	5z-6	–	–	–^*^	–	–	0.5^T^	–	–	±	±	–	–	–	–	–	–	–^*^	–	–	–
*Pichia kluyveri*	11/2–104	–	–	–	–	–	–	–	–^*^	1^T^	–	–	–	–	–	–	–	–^*^	–	–	–
*Pichia membranifaciens*	4/1–34	–	–	±	±	±	2^T^	3^T^	–	–	–	–	–	–	–	–	–	2^t^^*^	–	–	–
*Pichia scaptomyzae*	12z-4	–	–	–^*^+	–^*^+	–^*^+	1^T^	–	–	–^*^	–	–	–	–	–	–	–	0.5^t^^*^	–	–	–
*Saccharomyces cerevisiae*	14z-1	–	–	–^*^	–^*^	–^*^	0.5^T^	0.5	–^*^	–	–	–^*^	–^*^	–	–	–	–	2.5^t^^*^	–	–	–
*Saccharomyces paradoxus*	10z-2	–	–	–^*^	–^*^	–^*^	2	–	1^*^	–	–	–^*^	–	–	–	–	–	2^*^	–	–	–
*Saccharomyces uvarum*	4/2z-11	–	–	–	–^*^	–^*^	1	–	1.5^*^	–	±	–^*^	–	–	–	–	–	1^*^	–	–	–
*Torulaspora delbrueckei*	1/1z-10	–	–	–^*^	–^*^	–^*^	0.5	–	–^*^	–	±	–^*^	–	–	–	–	–	2^*^	–	–	–
*Wickerhamomyces anomalus*	15z-7	–	–	–	–	–	–	0.5	2^T^	3^T^	–	–	–	1	2	0.2	1	–	1^T^	–	–
*Zygoascus meyerae*	8z-2	–	–	–	–	–	0.5^T^	–	–^*^	–	–^*^^+^	–	–	–	–	–	–	1^t^^*^	–	–	–
*Zygosaccharomyces bailii*	4–24	–	–	–	–	–	1	0.5^T^	–^*^	–^*^	–	–^*^	–^*^	–	–	–	–	1^t^^*^	–	–	–
*Zygotorulaspora florentina*	7z–11	–	–	–	–	–	2^T^	–	–	–	–	–	–	–	–	–	–	–^*^	–	–	–

Numerals, width of the inhibition zone in mm; –, no interaction is detected; +, crossfeeding (stronger lawn growth around the yeast colony);

* , colony does not grow or grow poorly on the background lawn;

T *, the inhibition zone is turbid*.

When the isolates were tested for the assimilation of sugars as carbon sources (see above), an interesting mode of growth stimulation was noticed. On the plates supplemented with saccharose or melibiose, certain isolates able to utilize these disaccharides stimulated the growth of certain other isolates which were unable to assimilate them. An example is shown in Figure 2F. The print of the colony of the melibiose-negative isolate 11/2-104 (*Pichia kluyveri*) replica-plated on the melibiose medium grew along its edge facing the colony of the melibiose-positive isolate 7z–11 (*Zygotorulaspora florentina*). Apparently, the positive isolate supplied the negative isolate with utilizable carbon sources (crossfeeding).

### The effect of yeast isolates on the growth of *Botrytis*

**Figure 3:**
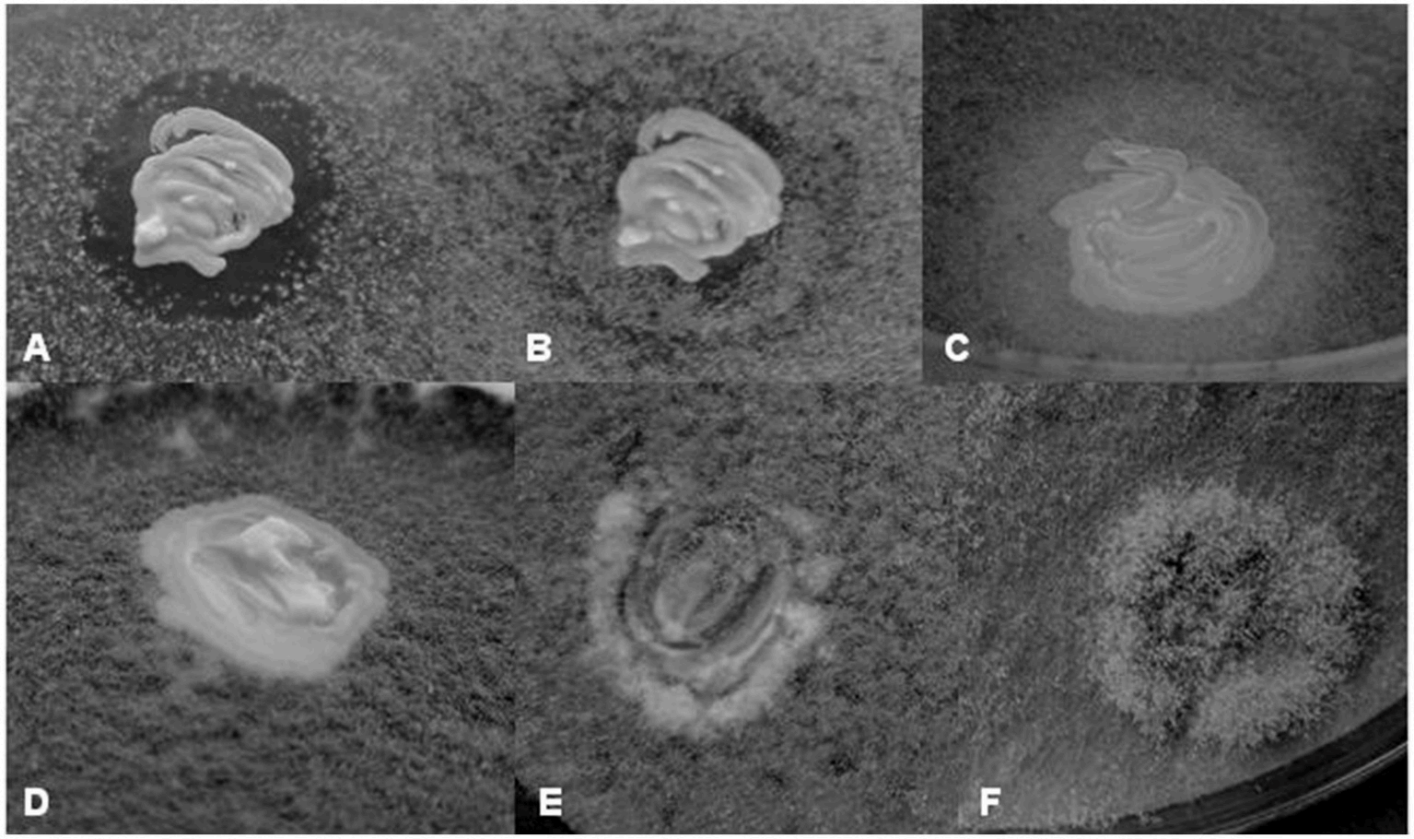
**Yeast-*Botrytis cinerea* interactions. (A)** Inhibition of the growth of *B. cinerea* around the *Metschnikowia* sp. 11/1–3 colony on YEA after 5 days of incubation. **(B)** Growth of the *Botrytis* mycelium into the inhibition zone after 11 days of incubation. Note that the contact with the yeast colony halts the growth of the mycelium. **(C)** Reduced mycelial growth around the *P. kluyveri* 11/2–104 colony on YEA after 5 days of incubation. **(D)** Contact inhibition: the *Botrytis* mycelium stops growing at the contact with the *P. fermentans* 5z-6 colony. **(E)** Gradual invasion of the *H. osmophila* 5/1z-3 colony by the *Botrytis* mycelium on YEA after 5 days of incubation. **(F)** Growth of the *Botrytis* mycelium on the *L. thermotolerans* 2/2z-8 colony. Note that the mycelium is thicker on the yeast colony.

The isolates selected for yeast-yeast interactions tests were also tested for effects on the growth of *B. cinerea*. Four types of interactions with the fungus were observed: (1) Clear inhibition zone around the yeast colony (Figure 3A), (2) turbid inhibition zone around the yeast colony (Figure 3C), (3) contact inhibition at the edge of the yeast colony (Figure 3D), and (4) overgrowth of the yeast colony by the *Botrytis* mycelium (Figures 3E,F). The clear zone can be interpreted as total inhibition of the growth of the fungus, whereas the turbid zone can be attributed to weaker inhibition or to competition for nutrients. The former morphology was observed only around *Metschnikowia, P. fermentans, Zygosaccharomyces*, and *Zygotorulaspora* isolates. Both types of zones and the contact inhibition (11 isolates) were visible only for 4 to 5 days, then the mycelium gradually grew into the zones (Figure 3B) and on the yeast colonies, indicating, that either the inhibitory conditions were changing with time or the hyphae invading the zones and the colonies were fed by cytoplasmic transport from the mycelium growing outside of the inhibition zone (e.g., Sipiczki, 2006). Representative results are shown in Table 4. Surprisingly, conspecificity did not always correlate with the intensity of the antagonistic activity. For species showing intraspecific diversity (*H. osmophila, H. vineae, Kl. dobzhanskii, L. thermotolerance, Metschnikowia sp., P. membranifaciens, S. cerevisiae, S. paradoxus, Zs. bailii, Zt. florentina*) more than one strain is listed in the table. Notably, more isolates produced zones on YEA than on PDA. The difference might be attributed to the much more vigorous growth of *Botrytis* on PDA.

**Table 4:** **Antagonistic effect of representative isolates of ascomyceteous yeast species on *B. cinerea***.

**Yeast**		***Botrytis cinerea***					
**Species**	**Isolate**	**Is inhibited around the yeast colony (inhibition zone in mm)**		**Grows onto the yeast colony**			
		**5 day**		**7 day**		**11 day**	
		**YEA**	**PDA**	**YEA**	**PDA**	**YEA**	**PDA**
*Candida glabrata*	14/1z-1	–	–	–	–	(+)	(+)
*Candida oleophila*	11/1–55	3^T^	–	–	–	(+)	–
*Hanseniaspora osmophila*	4–3	3^T^	–	+	+	+	+
	8–3	–	–	+	+	+	+
*Hanseniaspora uvarum*	9/1–66	5^T^	–	+	+	+	+
*Hanseniaspora vineae*	1/1–5	–	–	–	–	(+)	(+)
	11/1z-4	3^T^	–	+	–	+	+
	12/1z-2	3^T^	–	+	–	+	–
*Kluyveromyces dobzhanskii*	5z-9	1^T^	–	–	–	+	(+)
	9z-6	–	–	–	–	+	–
*Lachancea thermotolerans*	2/2z-8	–	–	+	+	+	+
	8/2z-3	4^T^	–	+	+	+	+
	14/1z-2	2^T^	–	+	+	+	+
*Metschnikowia* sp.	11/1–3	4	1	–	–	–	–
	15–8	3	–	–	–	–	–
*Pichia fermentans*	5z-6	4	4	–	–	(+)	–
*Pichia kluyveri*	11/2–104	5^T^	5^T^	–	–	(+)	–
*Pichia membranifaciens*	4/1–34	2^T^	–	(+)	–	+	(+)
	15/1z-9	2^T^	–	–	–	+	(+)
*Pichia scaptomyzae*	12z-4	–	–	(+)	–	+	(+)
*Saccharomyces cerevisiae*	3/1z-5	–	–	–	–	+	+
	14z-1	–	–	–	–	(+)	(+)
*Saccharomyces paradoxus*	3z-28	–	–	–	–	(+)	–
	7/2z-2	3^T^	–	–	–	+	+
	10z-2	3^T^	3^T^	–	–	(+)	–
*Saccharomyces uvarum*	4/2z-11	2^T^	2^T^	–	–	+	+
*Torulaspora delbrueckei*	1/1z-10	–	–	–	–	(+)	–
*Wickerhamomyces anomalus*	15z-7	–	–	–	–	–	(+)
*Zygoascus meyerae*	8z-2	4^T^	4^T^	–	–	+	–
*Zygosaccharomyces bailii*	4–24	3	3	–	–	(+)	(+)
	8–29	2	2	–	–	(+)	–
*Zygotorulaspora florentina*	2z-30	–	–	–	–	–	–
	3/2–1	–	–	+	(+)	+	+
	7z-11	3	1	–	–	(+)	–
	9/2z-9	3	1	–	–	(+)	(+)

YEA and PDA are media (see Materials and Methods for description); numerals, width of inhibition zone in mm; +, growth; (+), weak growth; –, no growth;

T *, inhibition zone is turbid*.

## Discussion

### Recovery of viable yeasts

When the grape is harvested, it is unavoidable, that berries fall to the ground and bunches remain on the vines. The former fall prey to rotting soil fungi and bacteria, which can also decompose the associated yeasts. The bunches on the vines are isolated from the destructive soil microorganisms and will be dehydrated (mummified) by frost and wind on the vines. The results of this study demonstrate, that fermentative yeasts relevant for wine making can tide over winter in these mummies.

This study was focused on the surviving yeasts, therefore the yeast communities of the overwintering grapes were examined by the conventional agar-plate method to obtain viable yeasts able to form colonies on a laboratory medium. The culture-independent strategies (e.g., metagenomics methods, DGGE) based on the analysis of DNA extracted directly from the yeast-containing substrates can identify yeast DNA but cannot distinguish between the DNA sequences of dead and live organisms (Mills et al., 2002; Prakitchaiwattana et al., 2004; Bokulich et al., 2014; Setati et al., 2015).

As certain yeasts of wine-making relevance are usually very sparse on grape berries if present at all (e.g., Mortimer and Polsinelli, 1999), an enrichment step was also included in the procedure. After plating out small aliquots on the agar plates, larger volumes of the samples were added to the enrichment medium restrictive for most non-*Saccharomyces* yeasts. By plating out aliquots before and after enrichment, large number of colonies were randomly isolated from 48 mummified grape bunches collected in 16 locations covering the entire Tokaj wine-growing region, (shared by Hungary and Slovakia; Figure 1) at the end of winter, shortly before pruning.

### Taxonomy of isolates

To broaden the spectrum of the phenotypic traits applicable to clustering of the isolates, the colonies were tested for the utilization of 14 compounds as carbon sources and lysine as a nitrogen source. The taxonomic affiliation of the phenotypic groups was then determined by sequencing the chromosomal segments coding for the D1/D2 domains of the LSU (large subunit) rRNA from randomly chosen isolates of the clusters.

Among the isolates, more ascomycetous species and fewer basidiomycetous species were identified but neither group was represented in all samples. All basidiomyceteous species are known phylloplane yeasts (for a review, see Fonseca and Inacio, 2006) belonging to Agaricomycotina or to Pucciniomycotina and have been detected on grape as well (e.g., Yanagida et al., 1992; Sabate et al., 2002; Raspor et al., 2006; Li et al., 2010; Cadez et al., 2010; Bourret et al., 2013; Lederer et al., 2013; Brysch-Herzberg and Seidel, 2015; Nemcová et al., 2015; Setati et al., 2015). The basidiomyceteous yeasts were not considered further in this study because of their marginal significance in winemaking.

The ascomycetevous yeasts most frequently detected in the mummified samples were *Metschnikowia* strains producing pigmented colonies and *Hanseniaspora* strains producing apiculate cells. The *Hanseniaspora* isolates were assigned to three species, *H. osmophila, H. uvarum*, and *H. vineae*, with *H. uvarum* being more frequent in samples directly spread on agar plates and *H. osmophila* being more frequent in the enriched cultures. The apiculate yeasts usually predominate the early phase of fermentation and produce compounds, that enrich the aroma profile of the wine (e.g., Zironi et al., 1993; Romano et al., 2003; Moreira et al., 2011). Here, *H. osmophila* was found in the enrichment cultures even if undetected in the non-enriched samples, indicating, that *H. osmophila* can better tolerate high alcohol concentrations than the other two species of the genus. The abundance of the *Hanseniaspora* cells in the samples demonstrates that these yeasts cope well with the harsh microclimatic conditions during overwintering.

Pulcherrimin-producing *Metschnikowia* strains are common on ripe grapes. They are usually assigned to *M. pulcherrima* (*C. pulcherrima*) or less frequently to *M. fructicola* in the oenological literature. However, the pigmented colonies isolated in this study differed from the type strains of all known pulcherrimin-producing species (*pulcherrima* clade) in their D1/D2 sequences and usually contained several ambiguous nucleotides. D1/D2 differences between grape-borne *Metschnikowia* strains and the type strains of the related species were already noticed in a previous study of Tokaj grape yeasts (Sipiczki, 2006). Recently, Brysch-Herzberg and Seidel (2015) encountered a similar problem with *Metschikowia* yeasts isolated from ripe wine grapes in Germany. These difficulties indicate, that the deficiency of the rDNA homogenization process recently discovered in *M. fructicola* and *M. andauensis* (Sipiczki et al., 2013) might characterize all pigmented *Metschnikowia* strains and obscure the species boundaries in the *pulcherrima* clade. As these species cannot be clearly separated by physiological tests either (Lachance, 2011), the taxonomic assignment of the *Metschnikowia* yeasts described in the oenological literature should be treated with prudence. Nevertheless, the isolates examined in this work undoubtedly belong to the *pulcherrima* clade which harbors the pigmented species of the genus (Lachance, 2011). Strains of the clade are usually present in the must during the early phase of fermentation. Their contribution to the quality of the wine is beyond doubt but not yet fully explored (e.g., Gil et al., 1996; Sadineni Naresh et al., 2012; Jolly et al., 2014; Contreras et al., 2015).

The other ascomyceteous yeast species were less abundant than *Metschnikowia* and *Hanseniaspora* in the sampled mummified bunches. Except for a few bunches, ascomyceteous non-*Metschnikowia* and non-*Hanseniaspora* yeasts were found only in the enrichment cultures. Interestingly, even species which are unable to utilize raffinose (*Ca. glabrata. Ca. oleophila, P. kluyvei, P. membranifaciens, T. delbruckei, Za. meyerae, Zs. bailii*) were enriched. In their case, the enriching factor could have been the high alcohol concentration of the medium lethal to *Metschnikowia*, most *Hanseniaspora* species, and the basidiomycetes.

The principal wine yeasts, S. *cerevisiae* and S. *uvarum*, are rarely isolated from grapes by conventional direct agar plating procedures, and there is an ongoing debate about their natural origin in wine fermentation (e.g., Fleet et al., 2002). *S. cerevisiae* was occasionally isolated from mature, overripe, and damaged grapes, but usually enrichment steps had to be applied, that elicit the recovery of minority species which would not be detected by direct plating (e.g., Mortimer and Polsinelli, 1999; Mercado et al., 2007; Sampaio and Goncalves, 2008; Peter et al., 2011). Consistent with these earlier observations, no *Saccharomyces* was found in this study among the colonies when the samples were plated directly on the agar medium. Upon enrichment, *S. paradoxus, S. cerevisiae*, and *S. uvarum* could be recovered from certain cultures. Remarkably, *S. paradoxus* was more frequent than the other *Saccharomyces* species. This finding is consistent with the reports on large distribution of *S. paradoxus* in certain grape-growing areas (Redzepovic et al., 2002) but inconsistent with the microbiological analyses which detected only *S. cerevisiae* and *S. uvarum* in fermenting Tokaj wines (Sipiczki et al., 2001; Naumov et al., 2002; Antunovics et al., 2003, 2005). Another interesting finding is the presence of *S. uvarum* in one of the overwintering populations because this yeast has not been reported yet from grape samples. These results unanimously prove, that *S. cerevisiae, S. paradoxus* and *S. uvarum* can participate in the colonization of grape berries and can also be transmitted in mummified grape berries over consecutive vegetation periods. Nevertheless, their rather sporadic occurrence indicates, that either they are not regular components of the colonizing yeast communities or they have poor winter tolerance, a property assumed to depend on the sporulation efficiency (Sipiczki, 2010). Further, experiments could reveal to what extent their survival in the mummified grape can contribute to the maintenance of the continuity of the *Saccharomyces* populations in vineyards. It is worth noting that the inability to utilize lysine as a nitrogen source was not an exclusive trait of *Saccharomyces* isolates in this study. Many isolates assigned to 6 other ascomyceteous and 2 basidiomyceteous species could not utilize lysine either. Thus, the widely used method of differentiation of *Saccharomyces* (lys^−^) and non-*Saccharomyces* yeasts (lys^+^) on the basis of lysine utilization (Angelo and Siebert, 1987) can lead to false results when not combined with other tests.

Surprisingly, no strains of *Ca. zemplinina* were found among the isolates although this osmotolerant and psychrotolerant species (Sipiczki, 2003) is quite regularly encountered on ripe grape (e.g., Li et al., 2010; Brežná et al., 2010; Cadez et al., 2010; Sun et al., 2014; Brysch-Herzberg and Seidel, 2015; Setati et al., 2015) and is the third major wine yeast in the Tokaj region (Csoma and Sipiczki, 2008). As the locations of sample collection covered the entire Tokaj region, the lack of *Ca. zemplinina* among the viable yeasts can be attributed to its inability to survive in the overwintering grapes rather than to its absence on the ripe grapes.

Certain grape samples yielded rapidly extending colonies of yeast-like cells fringed by wide hyphal halos. Their morphology suggested conspecificity with *A. pullulans*, a widespread phylloplane fungus (e.g., Grube et al., 2011) belonging to Pezizomycotina. Surprisingly, the D1/D2 sequences of the isolates indicated closer genetic affinity with *A. subglaciale, Ka. Microsticta*, and *Co. fagi* than with *A. pullulans*. *A. subglaciale* was described from subglacial ice (Zalar et al., 2008; Gostinčar et al., 2014) and has not yet been detected in the wine-related environment. As for the isolates showing 100% D1/D2 sequence identity with *Ka. microsticta* and *Co. fagi*, it is worth mentioning, that *Ka. microsticta* ITS sequences were recently amplified from grape must in South Africa (Setati et al., 2015). However, the similarity to database *Ka. microsticta* ITS sequences does not prove conspecificity with *Ka. microsticta* because no *Co. fagi* sequences are available in the databases. In addition, Setati et al. (2015) did not culture the strains from the samples. Thus, this is the first report on the isolation of *A. subglaciale* and *Ka. microsticta/Co. fagi* from grape. As these species are closely related and can easily be confused with *A. pullulans* (Zalar et al., 2008), earlier reports on the occurrence of *A. pullulans* on grapes should be taken with caution if not supported with adequate taxonomic analyses.

### Osmotolerance of isolates

Since, the yeasts residing in the overwintering grape berries have to cope with unfavorable factors, such as the antagonistic effects of other microbes and the osmotic pressure increasing during the dehydration of the berries, representatives of the ascomycetous yeast isolates were tested for response to these challenges. To investigate the ability of the isolates to cope with high osmotic pressure, representatives of the identified ascomyceteous species were tested for growth on agar plates supplemented with various concentrations of sugar. To mimic the real situation, glucose, and fructose were used in 1:1 proportion for supplementation. The *W. anomalus* isolates surpassed all other isolates in osmotolerance. This species has been described before as halophilic (Kagiyama et al., 1988) and a frequent spoilage yeast of fruit juice concentrates (e.g., Combina et al., 2008). As most isolates did not grow or poorly grew at 50% sugar, it can be assumed that the increasing osmotic pressure may also be involved in the preservation of the yeast community in the berries.

### Antagonistic and synergistic interactions

The interaction tests revealed both antagonistic (growth inhibition) and synergistic (growth promotion) interactions among the isolates. In the test method applied in this study, the lawn of the antagonistic isolate hampered the growth of the colony of the sensitive isolate inoculated on it. In the reversed situation, the colony of the antagonistic isolate elicited an inhibition zone around its colony in the lawn of the sensitive isolate. Turbid zones indicated milder antagonisms (reduction of growth in the sensitive lawn) probably attributable to the depletion of the medium of certain nutrients by the colony of the “antagonist” (competition for nutrients). Clear zones were produced when the antagonist caused total growth inhibition in the lawn of the sensitive isolate. Several mechanisms might account for total inhibition. One possibility is, that the antagonistic isolate killed the cells of the sensitive isolate by secreting a toxic agent into the medium. Numerous yeasts species have been found to have strains harboring extracellular genetic elements (killer factors) encoding secretable agents referred to as killer toxins (for a review, see Schmitt and Breinig, 2002). The clear zones in the lawn of certain non-*Saccharomyces* isolates around the *Saccharomyces* colonies might be caused by killer toxins. Testing the antagonistic isolates identified in this study for the presence of such killer factors in their cells will be the subject of a different study. Nevertheless, it is rather unlikely that the *Metschnikowia* isolates inhibited the growth of other yeasts by killer toxins. They all formed colonies fringed by maroon-red pigmented zones in the agar media. In a previous study (Sipiczki, 2006), the pigmented zones were found to coincide with the growth inhibition zones around the *Metschnikowia* colonies inoculated onto lawns of sensitive microorganisms. It turned out that the growth inhibition was due to the immobilization of the free iron in the medium by complexing the ferric ions with a compound secreted by the *Metschnikowia* cells (Sipiczki, 2006). The complex referred to as pulcherrimin is water-insoluble and has maroon-red color (Cook and Slater, 1956). The behavior of the *Wickerhamomyces* strain shown in Table 2 and Figure 2 demonstrates how complex the interactions within the yeast communities can be. It grew poorly on the lawn of most isolates, but generated clear inhibition zones in the lawn of many of them and its lawn was inhibited by the colonies of certain other isolates. This diversity of interactions is consistent with the diversity of modes by which *W. anomalus* (*P. anomala*) can antagonize other microorganisms. Its strains can produce inhibitory amounts of ethyl acetate (Fredlund et al., 2004), secrete killer toxins (Kagiyama et al., 1988), and cel-wall lytic enzymes (Jijakli and Lepoivre, 1998). Synergistic, growth-facilitating effects of certain isolates on other isolates were observed on all media used in this study and could be attributed to crossfeeding with nutrients. On media containing mellibiose [D-galactose-α(1 → 6)-D-glucose] as carbon sources, the melibiose-utilizing isolates (*Zs. florentina*) promoted the growth of the melibiose-minus (*P. kluyveri*) isolates. Most probably, the former hydrolyzed the disaccharide in excessive amount and released some of the monosaccharides utilizable by the latter into the medium. On the grapes similar synergistic interactions can take place but with different nutrients.

In the Tokaj region, grape is harvested late in the autumn after a long period of ripening during which high proportions of berries undergo noble rotting generated by the *B. cinerea* infection. The invasion of the berries by the hyphae of the fungus causes ruptures in the skin which are then colonized by yeasts and bacteria. It was found, that at least one type of the colonizing yeasts, the pulcherrimin-producing *Metschnikowia* strains can antagonize the growth of *Botrytis* by inhibiting the germination of its conidia and the extension of its hyphae (Sipiczki, 2006). Consistent with this observation, all *Metschnikowia* isolates investigated in this study showed anti-*Botrytis* antagonism manifested in clear inhibition zones in the mycelium around their colonies. As the inhibition zones and the pigmented halos coincided, it is likely that the growth inhibition by *Metschnikowia* was due to iron immobilization by a secreted compound as described above. The clear zones around *P. fermentans, Zs. Bailii*, and *Za. florentina* colonies are most probably due to different mechanisms because these yeasts do not produce pulcherrimin. The mild inhibition of the fungal growth by *S. paradoxus* and *S. uvarum* isolates is an unexpected result. As the zones around their colonies were turbid, the inhibition can be ascribed to competition for nutrients rather than to the secretion of agents having antifungal activities. Contact inhibition observed at the colonies of 11 isolates is a phenomenon which has been noticed in certain yeasts before this study but the mechanisms by which these yeasts exert their influence on the hyphae has not yet been understood (for a review, see Liu et al., 2013).

### Potential contribution of the mummified grapes to the maintenance of the vineyard yeast microflora

Taken together, the findings of this study demonstrate, that the grapes mummified on the vine can serve as a safe reservoir of fermentative yeasts, including *Saccharomyces*, and can transmit these yeasts between consecutive years in the vineyard. It can be reasonably assumed, that these yeasts may contribute to the maintenance of a complex vineyard yeast flora of wine-making relevance over years, together with those dispersed by insects (e.g., wasps, bees, *Drosophila*), and birds (Stevic, 1962; Francesca et al., 2012; Stefanini et al., 2012; Lam and Howell, 2015) visiting the ripening berries. Further, studies are needed to reveal the relative significance of these sources in the maintenance of the autochtonous vineyard yeast communities because soil is a rather unfavorable environment for yeast overwintering (Parle and Di Menna, 1966), the ROS-based antimicrobial defense system kills the ingested yeasts very fast in *Drosophila* (Hoang et al., 2015) the persistence of yeasts in bird cloacae has been shown to be very short (Francesca et al., 2012), the social wasp *Vesta crabro* (“European hornet” originally native only to Europe) recently found to harbor fermentative yeasts in guts (Francesca et al., 2012) is not common (or even absent) in large areas of the globe, where wine is produced. Moreover, certain yeast species were detected in the *V. crabro* guts after grape maturation, suggesting, that the wasps gathered those yeasts from the grapes rather than delivered them there. Mummified grapes can also be rare when modern harvesting technology is used.

## Author contributions

The author confirms being the sole contributor of this work and approved it for publication.

### Conflict of interest statement

The author declares that the research was conducted in the absence of any commercial or financial relationships that could be construed as a potential conflict of interest.

## References

[B1] Alvarez-Perez S. Herrera C. M. (2013). Composition, richness and nonrandom assembly of culturable bacterial-microfungal communities in floral nectar of Mediterranean plants. FEMS Microbiol. Ecol. 83, 685–699. 10.1111/1574-6941.12027 23057414

[B2] Angelo J. Siebert K. J. (1987). A new medium for the detection of wild strains in brewing culture yeast. J. Am. Soc. Brew. Chem. 45, 135–140.

[B3] Antunovics Z. Csoma H. Sipiczki M. (2003). Molecular and genetic analysis of the yeast flora of botrytized Tokaj wines. Bull. l'O.I.V. 76, 380–397.

[B4] Antunovics Z. Irinyi L. Sipiczki M. (2005).Combined application of methods to taxonomic identification of *Saccharomyces* strains in fermenting botrytized grape must. J. Appl. Microbiol. 98, 971–979. 10.1111/j.1365-2672.2005.02543.x 15752344

[B5] Barata A. Malfeito-Ferreira M. Loureiro V. (2012).The microbial ecology of wine grape berries. Int. J. Food Microbiol. 153, 243–259. 10.1016/j.ijfoodmicro.2011.11.025 22189021

[B6] Becher P. G. Flick G. Rozpędowska E. Schmidt A. Hagman A. Lebreton S.. (2012). Yeast, not fruit volatiles mediate *Drosophila melanogaster* attraction, oviposition and development. Funct. Ecol. 26, 822–828. 10.1111/j.1365-2435.2012.02006.x26776734

[B7] Bisson L. F. Joseph C. M. L. (2009). Yeasts, in Biology of Microorganisms on Grapes, in Must and in Wine, eds König H. Unden G. Fröhlich J. (Berlin; Heidelberg: Springer-Verlag), 45–60.

[B8] Bokulich N. A. Thorngate J. H. Richardson P. M. Mills D. A. (2014). Microbial biogeography of wine grapes is conditioned by cultivar, vintage, and climate. Proc. Natl. Acad. Sci. U.S.A. 111, E139–E148. 10.1073/pnas.1317377110 24277822 PMC3890796

[B9] Bourret T. B. Grove G. G. George J. Vandemark G. J. Henick-Kling T. Glawe D. A. (2013). Diversity and molecular determination of wild yeasts in a central Washington State vineyard. N. Am. Fungi 8, 1–32. 10.2509/naf2013.008.015

[B10] Brežná B. Zenišová K. Chovanová K. Chebeňová V. Kraková L. Kuchta T.. (2010). Evaluation of fungal and yeast diversity in Slovakian wine-related microbial communities. Antonie Van Leeuwenhoek 98, 519–529. 10.1007/s10482-010-9469-6 20556654

[B11] Brysch-Herzberg M. Seidel M. (2015). Yeast diversity on grapes in two German wine growing regions. Int. J. Food Microbiol. 214, 137–144. 10.1016/j.ijfoodmicro.2015.07.034 26292165

[B12] Cadez N. Zupan J. Raspor P. (2010). The effect of fungicides on yeast communities associated with grape berries. FEMS Yeast Res. 10, 619–630. 10.1111/j.1567-1364.2010.00635.x 20491940

[B13] Chavan P. Mane S. Kulkarni G. Shaikh S. Ghormade V. Nerkar D. P.. (2009). Natural yeast flora of different varieties of grapes used for winemaking in India. Food Microbiol. 26, 801–808. 10.1016/j.fm.2009.05.005 19835764

[B14] Ciani M. Mannazzu I. Marinangeli P. Clementi F. Martini A. (2004). Contribution of winery-resident *Saccharomyces cerevisiae* strains to spontaneous grape must fermentation. Antonie Van Leeuwenhoek 85, 159–164. 10.1023/B:ANTO.0000020284.05802.d7 15031657

[B15] Combina M. Daguerre C. Massera A. Mercado L. Sturm M. E. Ganga A.. (2008). Yeast identification in grape juice concentrates from Argentina. Lett. Appl. Microbiol. 46, 192–197. 10.1111/j.1472-765X.2007.02291.x 18069982

[B16] Combina M. Mercado L. Borgo P. Elia A. Jofré V. Ganga A.. (2005). Yeasts associated to Malbec grape berries from Mendoza, Argentina. Int. J. Food Microbiol. 98, 1055–1061. 10.1111/j.1365-2672.2005.02540.x 15836474

[B17] Contreras A. Curtin C. Varela C. (2015). Yeast population dynamics reveal a potential ‘collaboration’ between *Metschnikowia pulcherrima* and *Saccharomyces uvarum* for the production of reduced alcohol wines during Shiraz fermentation. Appl. Microbiol. Biotechnol. 99, 1885–1895. 10.1007/s00253-014-6193-6 25388943

[B18] Cook A. H. Slater C. A. (1956). The structure of pulcherrimin. J. Chem. Soc. 1956, 4133–4135. 10.1039/jr9560004133

[B19] Cordero-Bueso G. Arroyo T. Serrano A. Tello J. Aporta I. Vélez M. D.. (2011). Influence of the farming system and vine variety on yeast communities associated with grape berries. Int. J. Food Microbiol. 145, 132–139. 10.1016/j.ijfoodmicro.2010.11.040 21185102

[B20] Csoma H. Sipiczki M. (2008). Taxonomic reclassification of *Candida stellata* strains reveals frequent occurrence of *Candida zemplinina* in wine fermentation. FEMS Yeast Res. 8, 328–336. 10.1111/j.1567-1364.2007.00339.x 18179579

[B21] Di Maio S. Polizzotto G. Di Gangi E. Foresta G. Genna G. Verzera A.. (2012). Biodiversity of indigenous *Saccharomyces* populations from old wineries of South-Eastern Sicily (Italy): preservation and economic potential. PLoS ONE 7:e30428. 10.1371/journal.pone.0030428 22393353 PMC3290603

[B22] Dobzhansky T. Cooper D. M. Phaff H. J. Knapp E. P. Carson H. L. (1956). Differential attraction of species of *Drosophila* to different species of yeasts. Ecology 37, 544–550. 10.2307/1930178

[B23] Egli C. M. Edinger W. D. Mitrakul C. M. Henick-Kling T. (1998). Dynamics of indigenous and inoculated yeast populations and their effect on the sensory character of Riesling and Chardonnay wines. J. Appl. Microbiol. 85, 779–789. 10.1046/j.1365-2672.1998.00521.x 9830113

[B24] Fell J. W. Boekhout T. Fonseca A. Scorzetti G. Statzell-Tallman A. (2000). Biodiversity and systematics of basidiomycetous yeasts as determined by large subunit rDNA D1/D2/domain sequence analysis. Int. J. Syst. Bacteriol. 50, 1351–1371. 10.1099/00207713-50-3-1351 10843082

[B25] Fleet G. H. Prakitchaiwattana C. Beh A. L. Heard G. (2002). The yeast ecology of wine grapes, in Biodiversity and Biotechnology of Wine Yeast, ed Ciani M. (Kerala: Research Signpost), 1–17.

[B26] Fonseca A. Boekhout T. Fell J. W. (2011). *Cryptococcus* Vuillemin (1901), in The Yeasts - A Taxonomic Study, eds Kurtzman C. P. Fell J. W. Boekhout T. (London: Elsevier), 1661–1737.

[B27] Fonseca A. Inacio J. (2006). Phylloplane yeasts, in Biodiversity and Ecophysiology of Yeasts, eds Rosa C. Peter G. (Berlin; Heidelberg: Springer-Verlag), 263–301.

[B28] Francesca N. Canale D. E. Settanni L. Moschetti G. (2012). Dissemination of wine-related yeasts by migratory birds. Envir. Microbiol. Rep. 4, 105–112. 10.1111/j.1758-2229.2011.00310.x 23757236

[B29] Fredlund E. Druvefors U. A. Olstorpe M. N. Passoth V. Schnürer J. (2004). Influence of ethyl acetate production and ploidy level on the anti-mould activity of *Pichia anomala*. FEMS Microbiol. Lett. 238, 133–137. 10.1016/j.femsle.2004.07.027 15336413

[B30] Gadanho M. Libkind D. Sampaio J. P. (2006). Yeast diversity in the extreme acidic environments of the Iberian Pyrite Belt. Microb. Ecol. 52, 552–563. 10.1007/s00248-006-9027-y 17013554

[B31] Gil J. V. Mateo J. J. Jimenez M. Pastor A. Huerta T. (1996). Aroma compounds in wine as influenced by apiculate yeasts. J. Food Sci. 61, 1247–1249. 10.1111/j.1365-2621.1996.tb10971.x

[B32] Gilbert D. G. (1980). Dispersal of yeasts and bacteria by *Drosophila* in temperate rain forest. Oecologia 46, 135–137. 10.1007/BF00346979 28310639

[B33] Goddard M. R. Anfang N. Tang R. Gardner R. C. Jun C. (2010). A distinct population of *Saccharomyces cerevisiae* in New Zealand: evidence for local dispersal by insects and human-aided global dispersal in oak barrels. Environ. Microbiol. 12, 63–73. 10.1111/j.1462-2920.2009.02035.x 19691498

[B34] Gostinčar C. Ohm R. O. Kogej T. Sonjak S. Turk M. Zajc J.. (2014). Genome sequencing of four *Aureobasidium pullulans* varieties: biotechnological potential, stress tolerance, and description of new species. BMC Genomics 15:549. 10.1186/1471-2164-15-549 24984952 PMC4227064

[B35] Grube M. Schmid F. Berg G. (2011). Black fungi and associated bacterial communities in the phyllosphere of grapevine. Fungal. Biol. 115, 978–986. 10.1016/j.funbio.2011.04.004 21944210

[B36] Gutierrez A. R. Santamaria P. Epifanio S. Garijo P. Lopez R. (1999). Ecology of spontaneous fermentation in one winery during 5 consecutive years. Letts. Appl. Microbiol. 29, 411–415. 10.1046/j.1472-765X.1999.00657.x

[B37] Hamamoto M. Boekhout T. Nakase T. (2011). *Sporobolomyces* Kluyver and van Niel (1924), in The Yeasts - A Taxonomic Study, eds Kurtzman C. P. Fell J. W. Boekhout T. (London: Elsevier), 1929–1990.

[B38] Hoang D. Kopp A. Chandler J. A. (2015). Interactions between *Drosophila* and its natural yeast symbionts - Is *Saccharomyces cerevisiae* a good model for studying the fly-yeast relationship? PeerJ 3:e1116. 10.7717/peerj.1116 26336636 PMC4556146

[B39] Jijakli M. H. Lepoivre P. (1998). Characterization of an exo-b-1,3- glucanase produced by *Pichia anomala* strain K, antagonist of *Botrytis cinerea* on apples. Phytopathology 88, 335–343. 10.1094/PHYTO.1998.88.4.335 18944957

[B40] Jolly N. P. Varela C. Pretorius I. S. (2014). Not your ordinary yeast: non-*Saccharomyces* yeasts in wine production uncovered. FEMS Yeast Res. 14, 215–237. 10.1111/1567-1364.12111 24164726

[B41] Kagiyama S. Aiba T. Kadowaki K. Mogi K. (1988). New killer toxins of halophilic *Hansenula anomala*. Agric. Biol. Chem. 52, 1–7. 10.1271/bbb1961.52.1

[B42] Kwon-Chung K. J. (2011). *Filobasidium* Olive (1968), in The Yeasts - A Taxonomic Study, eds Kurtzman C. P. Fell J. W. Boekhout T. (London: Elsevier), 1457–1465.

[B43] Lachance M. A. (2011). Metschnikowia Kamienski (1899), in The Yeasts - A Taxonomic Study, eds Kurtzman C. P. Fell J. W. Boekhout T. (London: Elsevier), 575–619.

[B44] Lachance M. A. Gilbert D. G. Starmer W. T. (1995). Yeast communities associated with *Drosophila* species and related flies in an eastern oak-pine forest: a comparison with western communities. J. Ind. Microbiol. 14, 484–494. 10.1007/BF01573963 7662291

[B45] Lam S. S. T. H. Howell K. S. (2015). *Drosophila*-associated yeast species in vineyard ecosystems. FEMS Microbiol. Lett. 362:fnv170. 10.1093/femsle/fnv170 26391524

[B46] Lederer M. A. Nielsen D. S. Toldam-Andersen T. B. Herrmann J. V. Arneborg N. (2013). Yeast species associated with different wine grape varieties in Denmark. Acta Agric. Scand. B Soil Plant Sci. 63, 89–96. 10.1080/09064710.2012.723738

[B47] Li S. S. Cheng C. Li Z. Chen J. Y. Yan B. Han B. Z.. (2010). Yeast species associated with wine grapes in China. Int. J. Food Microbiol. 138, 85–90. 10.1016/j.ijfoodmicro.2010.01.009 20116124

[B48] Liu J. Sui Y. Wisniewski M. Droby S. Liu Y. (2013). Review: utilization of antagonistic yeasts to manage postharvest fungal diseases of fruit. Int. J. Food Microbiol. 167, 153–160. 10.1016/j.ijfoodmicro.2013.09.004 24135671

[B49] Loureiro V. Malfeito-Ferreira M. (2003). Spoilage yeasts in the wine industry. Int. J. Fodd Microbiol. 86, 23–50. 10.1016/S0168-1605(03)00246-0 12892920

[B50] Magyar I. Bene Z. (2004). Characterization of yeast and mould biota of botrytized grapes in Tokaj wine region in the years 2000 and 2001. Acta Alimentaria 3, 259–267. 10.1556/AAlim.33.2004.3.6

[B51] Martini A. (1993). The origin and domestication of the wine yeast *Saccharomyes cerevisiae*. J. Wine Res. 4, 165–176. 10.1080/09571269308717966

[B52] Mercado L. Dalcero A. Masuelli R. Combina M. (2007). Diversity of *Saccharomyces* strains on grapes and winery surfaces: analysis of their contribution to fermentative flora of Malbec wine from Mendoza (Argentina) during two consecutive years. Food Microbiol. 24, 403–412. 10.1016/j.fm.2006.06.005 17189766

[B53] Mills D. A. Eric J. Eric A. Cocolin L. (2002). Yeast diversity and persistence in *Botrytis*-affected wine fermentation. Appl. Environ. Microbiol. 68, 4884–4893. 10.1128/AEM.68.10.4884-4893.2002 12324335 PMC126389

[B54] Morais P. B. Lachance M.-A. Rosa C. A. (2005). Saturnispora hagleri sp. nov., a yeast species isolated from Drosophila flies in Atlantic rainforest in Brazil. Int. J. System. Evol. Microbiol. 55, 1725–1727. 10.1099/ijs.0.63673-0 16014509

[B55] Moreira N. Pina C. Mendes F. Couto J. A. Hogg T. Vasconcelos I. (2011). Volatile compounds contribution of *Hanseniaspora guilliermondii* and *Hanseniaspora uvarum* during red wine vinifications. Food Control 22, 662–667. 10.1016/j.foodcont.2010.07.025

[B56] Mortimer R. Polsinelli M. (1999). On the origins of wine yeast. Res. Microbiol. 150, 199–204. 10.1016/S0923-2508(99)80036-9 10229949

[B57] Naumov G. I. Naumova E. S. Antunovics Z. Sipiczki M. (2002). *Saccharomyces bayanus* var. uvarum in Tokaj wine-making of Slovakia and Hungary. Appl. Microbiol. Biotechnol. 59, 727–730. 10.1007/s00253-002-1077-6 12226732

[B58] Nemcová K. Breierová E. Vadkertiová R. Molnárová J. (2015). The diversity of yeasts associated with grapes and musts of the Strekov winegrowing region, Slovakia. Folia Microbiol. 60, 103–109. 10.1007/s12223-014-0347-x 25253264

[B59] Palanca L. Gaskett A. C. Günther C. S. Newcomb R. D. Goddard M. R. (2013). Quantifying variation in the ability of yeasts to attract *Drosophila melanogaster*. PLoS ONE 8:e75332. 10.1371/journal.pone.0075332 24086510 PMC3783394

[B60] Parle J. N. Di Menna M. E. (1966). The source of yeasts in New Zealand wines. N.Z. J. Agric. Res. 9, 98–107. 10.1080/00288233.1966.10418122

[B61] Peter G. Dlauchy D. Szucs E. Tornai-Lehoczki J. (2011). Enrichment in methanol containing broth – a simple method for the isolation of *Saccharomyces* from grapes. Acta Alimentaria 40, 376–384. 10.1556/AAlim.40.2011.3.8

[B62] Phaff H. J. Knapp E. P. (1956). The taxonomy of yeasts found in exudates of certain trees and other natural breeding sites of some species of *Drosophila*. Antonie Van Leeuwenhoek 22, 117–130. 10.1007/BF02538319 13340698

[B63] Prakitchaiwattana C. J. Fleet G. H. Heard G. M. (2004). Application and evaluation of denaturing gradient gel electrophoresis to analyse the yeast ecology of wine grapes. FEMS Yeast Res. 4, 865–877. 10.1016/j.femsyr.2004.05.004 15450194

[B64] Raspor P. Milek D. M. Polanc J. Mozina S. S. Cadez N. (2006). Yeasts isolated from three varieties of grapes cultivated in different locations of the Dolenjska vine-growing region, Slovenia. Int. J. Food Microbiol. 109, 97–102. 10.1016/j.ijfoodmicro.2006.01.017 16626833

[B65] Redzepovic S. Orlic S. Sikora S. Majdak A. Pretorius I. S. (2002). Identification and characterization of *Saccharomyces cerevisiae* and *Saccharomyces paradoxus* strains isolated from Croatian vineyards. Lett. Appl. Microbiol. 35, 305–310. 10.1046/j.1472-765X.2002.01181.x 12358693

[B66] Renouf V. Claisse O. Lonvaud-Funel A. (2007). Inventory and monitoring of wine microbial consortia. Appl. Microbiol. Biotechnol. 75, 149–164. 10.1007/s00253-006-0798-3 17235561

[B67] Romano P. Fiore C. Paraggio M. Caruso M. Capece A. (2003). Function of yeast species and strains in wine flavour. Int. J. Food Microbiol. 86, 169–180. 10.1016/S0168-1605(03)00290-3 12892932

[B68] Rosini G. (1984). Assessment of dominance of added yeast with fermentation and origin of *Saccharomyces cerevisiae* in wine making. J. Gen. Appl. Microbiol. 30, 249–256. 10.2323/jgam.30.249

[B69] Sabate J. Cano J. Esteve-Zarzoso B. Guillamón J. M. (2002). Isolation and identification of yeasts associated with vineyard and winery by RFLP analysis of ribosomal genes and mitochondrial DNA. Microbiol. Res. 157, 267–274. 10.1078/0944-5013-00163 12501990

[B70] Sadineni Naresh V. Kondapalli N. Obulam V. (2012). Effect of co-fermentation with *Saccharomyces cerevisiae* and *Torulaspora delbrueckii* or *Metschnikowia pulcherrima* on the aroma and sensory properties of mango wine. Ann. Microbiol. 62, 1353–1360. 10.1007/s13213-011-0383-6

[B71] Sampaio J. P. (2011a). *Curvibasidium* Sampaio and Golubev (2004), in The Yeasts - A Taxonomic Study, eds Kurtzman C. P. Fell J. W. Boekhout T. (London: Elsevier), 1413–1418.

[B72] Sampaio J. P. (2011b). *Rhodotorula* Harrison (1928), in The Yeasts - A Taxonomic Study, eds Kurtzman C. P. Fell J. W. Boekhout T. (London: Elsevier), 1873–1927.

[B73] Sampaio J. P. Goncalves P. (2008). Natural populations of *Saccharomyces kudriavzevii* in Portugal are associated with oak bark and are sympatric with *S. cerevisiae and S. paradoxus*. Appl. Environ. Microbiol. 74, 2144–2152. 10.1128/AEM.02396-07 18281431 PMC2292605

[B74] Santamarıa P. Garijo P. Lopez R. Tenorio C. Rosa Gutierrez A. (2005). Analysis of yeast population during spontaneous alcoholic fermentation: effect of the age of the cellar and the practice of inoculation. Int. J. Food Microbiol. 103, 49–56. 10.1016/j.ijfoodmicro.2004.11.024 16084265

[B75] Schmitt M. J. Breinig F. (2002). The viral killer system in yeast: from molecular biology to application. FEMS Microbiol. Rev. 748, 1–20. 10.1111/j.1574-6976.2002.tb00614.x 12165427

[B76] Schuller D. Alves H. Dequin S. Casal M. (2005). Ecological survey of *Saccharomyces cerevisiae* strains from vineyards in the Vinho Verde region of Portugal. FEMS Microbiol. Ecol. 51, 167–177. 10.1016/j.femsec.2004.08.003 16329865

[B77] Scorzetti G. Fell J. W. Fonseca A. Statzell-Tallman A. (2002). Systematics of basidiomycetous yeasts: a comparison of large subunit D1/D2 and internal transcribed spacer rDNA regions. FEMS Yeast Res. 2, 495–517. 10.1111/j.1567-1364.2002.tb00117.x 12702266

[B78] Setati M. E. Jacobson D. Andong U.-C. Bauer F. (2012). The vineyard yeast microbiome, a mixed model microbial map. PLoS ONE 7:e52609. 10.1371/journal.pone.0052609 23300721 PMC3530458

[B79] Setati M. E. Jacobson D. Bauer F.F. (2015). Sequence-based analysis of the Vitis vinifera L. cv Cabernet Sauvignon grape must mycobiome in three South African vineyards employing distinct agronomic systems. Front. Microbiol. 6:1358. 10.3389/fmicb.2015.01358 26648930 PMC4663253

[B80] Sipiczki M. (2003). Candida zemplinina sp. nov., an osmotolerant and psychrotolerant yeast that ferments sweet botrytised wines. Int. J. Syst. Evol. Microbiol. 53, 2079–2083. 10.1099/ijs.0.02649-0 14657149

[B81] Sipiczki M. (2006). *Metschnikowia* strains isolated from botrytised grapes antagonize fungal and bacterial growth by iron depletion. Appl. Environ. Microbiol. 72, 6716–6724. 10.1128/AEM.01275-06 17021223 PMC1610289

[B82] Sipiczki M. (2010). Diversity, variability and fast adaptive evolution of the wine yeast (*Saccharomyces cerevisiae*) genome. Ann. Microbiol. 61, 85–93. 10.1007/s13213-010-0086-4

[B83] Sipiczki M. Pfliegler W. P. Holb I. J. (2013). *Metschnikowia* species share a pool of diverse rRNA genes differing in regions that determine hairpin-loop structures and evolve by reticulation. PLoS ONE 8:e67384. 10.1371/journal.pone.0067384 23805311 PMC3689696

[B84] Sipiczki M. Romano P. Lipani G. Miklos I. Antunovics Z. (2001). Analysis of yeasts derived from natural fermentation in a Tokaj winery. Antonie Van Leeuwenhoek 79, 97–105. 10.1023/A:1010249408975 11392490

[B85] Starmer W. T. Heed W. B. Miranda M. Miller M. W. Phaff H. J. (1976). The ecology of yeast flora associated with cactiphilic *Drosophila* and their host plants in the Sonoran desert. Microb. Ecol. 3, 11–30. 10.1007/BF02011450 24233393

[B86] Stefanini I. Dapporto L. Legras J.-L. Antonio Calabretta A. Di Paola M. De Filippo C.. (2012). Role of social wasps in *Saccharomyces cerevisiae* ecology and evolution. Proc. Natl. Acad. Sci. U.S.A. 109, 13398–13403. 10.1073/pnas.1208362109 22847440 PMC3421210

[B87] Stevic B. (1962). The importance of bees (*Apis* sp) and wasps (*Vespa* sp) as carrier of yeasts for the microflora of grapes and the quality of wines. Archiv Poljoprivredre Nauke Beograd 15, 80–91.

[B88] Sun Y. Guo J. Liu F. Liu Y. (2014). Identification of indigenous yeast flora isolated from the five winegrape varieties harvested in Xiangning, China. Antonie Van Leeuwenhoek 105, 533–540. 10.1007/s10482-013-0105-0 24395034

[B89] Taylor M. W. Tsai P. Anfang N. Ross H. A. Goddard M. R. (2014). Pyrosequencing reveals regional differences in fruit-associated fungal communities. Environ. Microbiol. 16, 2848–2858. 10.1111/1462-2920.12456 24650123 PMC4257574

[B90] Treseder K. K. Lennon J. T. (2015). Fungal traits that drive ecosystem dynamics on land. Microbiol. Mol. Biol. Rev. 79, 243–262. 10.1128/MMBR.00001-15 25971588 PMC4429240

[B91] Valero E. Cambon B. Schuller D. Casal M. Dequin S. (2007). Biodiversity of *Saccharomyces* yeast strains from grape berries of wine producing areas using starter commercial yeasts. FEMS Yeast Res. 7, 317–329. 10.1111/j.1567-1364.2006.00161.x 17040482

[B92] Vaughan-Martini A. Martini A. (1995). Facts, myths and legends on the prime industrial microorganisms. J. Indus. Microbiol. 14, 514–522. 10.1007/BF01573967 7662293

[B93] Yanagida F. Ichinose F. Shinohara T. Goto S. (1992). Distribution of wild yeasts in the white grape varieties at Central Japan. J. Gen. Appl. Microbiol. 38, 505–509. 10.2323/jgam.38.501

[B94] Zahavi T. Droby S. Cohen L. Weiss B. Ben-Arie R. (2002). Characterisation of the yeast flora on the surface of grape berries in Israel. Vitis 41, 203–308.

[B95] Zalar P. Gostinčar C. de Hoog G.S. Uršiè V. Sudhadham M. Gunde-Cimerman N. (2008). Redefinition of *Aureobasidium pullulans* and its varieties. Stud. Mycol. 61, 21–38. 10.3114/sim.2008.61.02 19287524 PMC2610310

[B96] Zironi R. Romano P. Suzzi G. Battistutta F. Comi G. (1993). Volatile metabolites produced in wine by mixed and sequential cultures of *Hanseniaspora guilliermondii* or *Kloeckera apiculata* and *Saccharomyces cerevisiae*. Biotechnol. Lett. 15, 235–238. 10.1007/BF0012831117440687

