# Supplementary material for: Overwintering of Vineyard Yeasts: Survival of Interacting Yeast Communities in Grapes Mummified on Vines
Source: Front Microbiol. 2016 Feb 29;7:212. doi: 10.3389/fmicb.2016.00212 (PMC4770031; doi:10.3389/fmicb.2016.00212)
Supplement: Supplementary file 1 [file Table1.DOC]

**TABLE S1 | The occurrence and relative abundance of the ascomyceteous species in the grape samples**

| **Sample** | **Enrichment** | **% among isolates** | | | | | | | | | | | | | | | | | | | | | | | |
| --- | --- | --- | --- | --- | --- | --- | --- | --- | --- | --- | --- | --- | --- | --- | --- | --- | --- | --- | --- | --- | --- | --- | --- | --- | --- |
|  |  | **Pezizo-**  **mycotina** | | **Saccharomycotina** | | | | | | | | | | | | | | | | | | | | | **Total** |
|  |  | ***A. subglaciale*** | ***Ka. microsticta*** | ***Ca. glabrata*** | ***Ca. oleophila*** | ***H. osmophila*** | ***H. uvarum*** | ***H. vinae*** | ***Kl. dobzhanskii*** | ***Kr. fluxuum*** | ***L. thermotolerans*** | ***Metschnikovia*** | ***P. fermentans*** | ***P. kluyveri*** | ***P. membranifaciens*** | ***P. scaptomyzae*** | ***S. cerevisiae*** | ***S. paradoxus*** | ***S. uvarum*** | ***T. delrueckii*** | ***W. anomalus*** | ***Za. meyerae*** | ***Zs. bailii*** | ***Zt. florentina*** |  |
| 1 | - |  |  |  |  |  | 78 |  |  |  |  | 22 |  |  |  |  |  |  |  |  |  |  |  |  | 100 |
|  | + |  |  |  | 5 |  | 14 |  |  |  |  | 81 |  |  |  |  |  |  |  |  |  |  |  |  | 100 |
| 1/1 | - |  |  |  |  |  | 59 | 37 |  |  |  | 3 |  |  |  |  |  |  |  | 1 |  |  |  |  | 100 |
|  | + |  |  |  |  | 41 |  |  |  |  |  | 39 |  |  |  |  |  |  |  | 20 |  |  |  |  | 100 |
| 1/2 | - |  | 77 |  |  |  |  |  |  |  |  | 14 |  |  |  |  |  |  |  |  |  |  |  |  | 91 |
|  | + |  |  |  |  |  |  |  |  |  |  |  |  |  |  |  |  |  |  |  |  |  |  |  | 0 |
| 2 | - |  | 33 |  |  |  | 67 |  |  |  |  |  |  |  |  |  |  |  |  |  |  |  |  |  | 100 |
|  | + |  |  |  |  | 8 |  |  |  |  |  |  |  |  |  |  |  |  |  |  |  |  |  | 92 | 100 |
| 2/1 | - |  |  |  |  |  |  |  |  |  |  |  |  |  |  |  |  |  |  |  |  |  |  |  | 0 |
|  | + |  |  |  |  |  |  |  |  |  |  | 100 |  |  |  |  |  |  |  |  |  |  |  |  | 100 |
| 2/2 | - |  |  |  |  |  |  |  |  |  |  |  |  |  |  |  |  |  |  |  |  |  |  |  | 0 |
|  | + |  |  |  |  |  |  |  |  |  | 33 | 67 |  |  |  |  |  |  |  |  |  |  |  |  | 100 |
| 3 | - |  |  |  |  |  | 7 |  |  |  |  | 70 |  |  |  |  |  |  |  |  |  |  |  |  | 77 |
|  | + |  |  |  |  |  |  |  |  |  |  | 96 |  |  |  |  |  | 4 |  |  |  |  |  |  | 100 |
| 3/1 | - |  |  |  |  |  |  |  |  |  |  | 64 |  |  |  |  |  |  |  |  |  |  |  | 36 | 100 |
|  | + |  |  |  |  |  |  |  |  |  |  | 52 |  |  |  |  | 48 |  |  |  |  |  |  |  | 100 |
| 3/2 | - |  |  |  |  |  |  | 17 |  |  |  | 17 |  |  |  |  |  |  |  |  |  |  |  | 66 | 100 |
|  | + |  |  |  |  |  |  |  |  |  |  |  |  |  |  |  |  | 64 |  |  |  |  |  | 36 | 100 |
| 4 | - |  |  |  |  | 33 |  |  |  |  |  | 61 |  |  |  |  |  |  |  |  |  |  | 6 |  | 100 |
|  | + |  |  |  |  | 42 |  |  |  |  |  | 58 |  |  |  |  |  |  |  |  |  |  |  |  | 100 |
| 4/1 | - |  |  |  |  |  | 16 |  |  |  |  | 44 |  |  | 7 |  |  |  |  |  |  |  |  |  | 67 |
|  | + |  |  |  |  |  |  |  |  |  |  |  |  |  | 100 |  |  |  |  |  |  |  |  |  | 100 |
| 4/2 | - | 17 |  |  |  |  | 70 |  |  |  |  | 11 | 2 |  |  |  |  |  |  |  |  |  |  |  | 100 |
|  | + |  |  |  |  |  | 8 |  |  |  |  |  |  |  |  |  |  |  | 8 |  |  |  |  | 84 | 100 |
| 5 | - |  |  |  |  |  | 24 |  |  |  |  | 21 |  |  |  |  |  |  |  |  |  |  |  |  | 45 |
|  | + |  |  |  |  |  |  |  | 23 |  |  | 6 | 65 |  |  |  |  | 6 |  |  |  |  |  |  | 100 |
| 5/1 | - |  |  |  |  |  |  |  |  |  |  | 75 |  |  |  |  |  |  |  |  |  |  |  |  | 75 |
|  | + |  |  |  |  |  | 56 |  |  |  | 31 | 13 |  |  |  |  |  |  |  |  |  |  |  |  | 100 |
| 5/2 | - |  |  |  |  | 77 |  |  |  |  |  | 17 |  |  |  |  |  |  |  |  |  |  |  |  | 94 |
|  | + |  |  |  |  | 73 |  |  |  |  |  | 27 |  |  |  |  |  |  |  |  |  |  |  |  | 100 |
| 6 | - |  |  |  |  |  |  |  |  |  |  |  |  |  |  |  |  |  |  |  |  |  |  |  | 0 |
|  | + |  |  |  |  |  |  |  |  |  |  |  |  |  |  |  |  |  |  |  |  |  |  |  | 0 |
| 6/1 | - |  | 100 |  |  |  |  |  |  |  |  |  |  |  |  |  |  |  |  |  |  |  |  |  | 100 |
|  | + |  |  |  |  |  |  |  |  |  |  |  |  |  |  |  |  |  |  |  |  |  |  |  | 0 |
| 6/2 | - |  | 7 |  |  |  |  |  |  |  |  |  |  |  |  |  |  |  |  |  |  |  |  |  | 7 |
|  | + |  |  |  |  |  |  |  |  |  |  |  |  |  |  |  |  |  |  |  |  |  |  |  | 0 |
| 7 | - |  |  |  |  |  | 32 |  |  | 14 |  | 35 |  |  |  |  |  |  |  |  |  |  |  |  | 81 |
|  | + |  |  |  |  | 94 |  |  |  |  |  |  |  |  |  |  |  |  |  |  |  |  |  | 6 | 100 |
| 7/1 | - |  |  |  |  |  |  |  |  |  |  | 5 |  |  |  |  |  |  |  |  |  |  |  |  | 5 |
|  | + |  |  |  |  |  |  |  | 100 |  |  |  |  |  |  |  |  |  |  |  |  |  |  |  | 100 |
| 7/2 | - |  |  |  |  |  | 77 |  |  |  |  | 7 |  |  |  |  |  |  |  |  |  |  |  |  | 84 |
|  | + |  |  |  |  | 25 |  |  |  |  |  |  |  |  |  |  |  | 75 |  |  |  |  |  |  | 100 |
| 8 | - |  |  |  |  | 56 |  |  |  | 7 |  | 33 |  |  |  |  |  |  |  |  |  |  | 4 |  | 100 |
|  | + |  |  |  |  | 51 |  |  |  |  | 24 |  |  |  |  |  |  |  |  |  |  | 25 |  |  | 100 |
| 8/1 | - |  |  |  |  | 47 |  |  |  |  |  | 36 |  |  |  |  |  |  |  |  |  |  |  |  | 83 |
|  | + |  |  |  |  |  |  |  |  |  |  | 100 |  |  |  |  |  |  |  |  |  |  |  |  | 100 |
| 8/2 | - |  |  |  |  |  | 42 |  |  |  | 47 | 8 |  |  |  |  |  |  |  |  |  |  |  |  | 97 |
|  | + |  |  |  |  |  |  |  |  |  | 100 |  |  |  |  |  |  |  |  |  |  |  |  |  | 100 |
| 9 | - |  |  |  |  |  | 19 |  |  |  |  |  |  |  |  |  |  |  |  |  |  |  |  |  | 19 |
|  | + |  |  |  |  | 26 |  |  | 74 |  |  |  |  |  |  |  |  |  |  |  |  |  |  |  | 100 |
| 9/1 | - |  |  |  |  |  | 16 |  |  |  | 19 | 33 |  |  |  |  |  |  |  |  |  |  |  |  | 68 |
|  | + |  |  |  |  |  |  |  |  |  | 91 | 9 |  |  |  |  |  |  |  |  |  |  |  |  | 100 |
| 9/2 | - |  |  |  |  |  |  |  |  |  |  | 100 |  |  |  |  |  |  |  |  |  |  |  |  | 100 |
|  | + |  |  |  |  | 11 |  |  | 45 |  |  |  |  |  |  |  |  |  |  |  |  |  |  | 44 | 100 |
| 10 | - | 8 |  |  |  |  |  |  |  |  |  | 20 |  |  |  |  |  |  |  |  |  |  |  |  | 28 |
|  | + |  |  |  |  |  |  |  | 24 |  |  | 5 |  |  |  |  |  | 71 |  |  |  |  |  |  | 100 |
| 10/1 | - |  |  |  |  |  |  |  |  |  |  |  |  |  |  |  |  |  |  |  |  |  |  |  | 0 |
|  | + |  |  |  |  |  |  |  | 100 |  |  |  |  |  |  |  |  |  |  |  |  |  |  |  | 100 |
| 10/2 | - |  |  |  |  |  |  |  |  |  |  |  |  |  |  |  |  |  |  |  |  |  |  |  | 0 |
|  | + |  |  |  |  |  |  |  |  |  | 100 |  |  |  |  |  |  |  |  |  |  |  |  |  | 100 |
| 11 | - |  |  |  |  |  | 43 |  |  |  | 11 | 18 |  |  |  |  |  |  |  |  |  | 14 |  |  | 86 |
|  | + |  |  |  |  |  |  |  |  |  | 100 |  |  |  |  |  |  |  |  |  |  |  |  |  | 100 |
| 11/1 | - |  |  |  | 29 |  | 56 |  |  |  |  | 12 |  |  |  |  |  |  |  |  |  |  |  |  | 97 |
|  | + |  |  |  | 71 |  |  | 29 |  |  |  |  |  |  |  |  |  |  |  |  |  |  |  |  | 100 |
| 11/2 | - |  |  |  |  |  | 52 |  |  |  |  | 22 |  |  |  |  |  |  |  |  |  |  |  |  | 74 |
|  | + |  |  |  |  | 57 |  |  |  |  | 43 |  |  |  |  |  |  |  |  |  |  |  |  |  | 100 |
| 12 | - |  |  |  |  |  | 34 |  |  |  |  | 8 |  |  |  |  |  |  |  |  |  |  |  |  | 42 |
|  | + |  |  |  |  |  |  |  |  |  |  |  |  |  |  | 100 |  |  |  |  |  |  |  |  | 100 |
| 12/1 | - |  |  |  |  |  |  | 19 |  |  |  | 5 |  |  |  |  |  |  |  |  |  | 2 |  |  | 26 |
|  | + |  |  |  |  |  |  | 39 |  |  | 61 |  |  |  |  |  |  |  |  |  |  |  |  |  | 100 |
| 12/2 | - |  |  |  |  | 35 | 47 |  |  |  |  |  |  |  |  |  |  |  |  |  |  |  |  |  | 82 |
|  | + |  |  |  |  | 60 |  |  |  |  | 40 |  |  |  |  |  |  |  |  |  |  |  |  |  | 100 |
| 13 | - |  | 40 |  |  |  |  |  |  |  |  |  |  |  |  |  |  |  |  |  |  |  |  |  | 40 |
|  | + |  |  |  |  |  |  |  |  |  |  |  |  |  |  |  |  |  |  |  |  |  |  |  | 0 |
| 13/1 | - |  | 5 |  |  |  |  |  |  |  |  |  |  |  |  |  |  |  |  |  |  |  |  |  | 5 |
|  | + |  |  |  |  |  |  |  |  |  | 75 |  |  |  |  |  |  |  |  |  |  |  |  |  | 75 |
| 13/2 | - |  |  |  |  |  | 48 |  |  |  |  | 26 |  |  |  |  |  |  |  |  |  |  |  |  | 74 |
|  | + |  |  |  |  | 29 |  |  |  |  | 14 |  |  |  |  |  |  |  |  |  |  |  |  | 57 | 100 |
| 14 | - |  |  |  |  |  | 33 | 17 |  |  |  | 25 |  |  |  |  |  |  |  |  |  |  |  |  | 75 |
|  | + |  |  |  |  | 14 |  |  |  |  | 43 |  |  |  |  |  | 43 |  |  |  |  |  |  |  | 100 |
| 14/1 | - |  |  |  |  |  | 24 |  |  |  |  | 24 |  |  |  |  |  |  |  |  |  |  |  |  | 48 |
|  | + |  |  | 25 |  |  |  |  |  |  | 75 |  |  |  |  |  |  |  |  |  |  |  |  |  | 100 |
| 14/2 | - |  |  |  |  |  |  |  |  |  |  | 100 |  |  |  |  |  |  |  |  |  |  |  |  | 100 |
|  | + |  |  |  |  |  |  |  |  |  | 100 |  |  |  |  |  |  |  |  |  |  |  |  |  | 100 |
| 15 | - |  |  |  |  |  |  |  |  |  |  | 36 |  |  |  |  |  |  |  |  |  |  |  |  | 36 |
|  | + |  |  |  |  |  |  |  |  |  |  | 55 |  |  |  |  |  |  |  |  |  | 45 |  |  | 100 |
| 15/1 | - |  |  |  |  |  | 84 |  |  |  |  | 2 |  |  | 8 |  |  |  |  |  |  |  |  |  | 94 |
|  | + |  |  |  |  |  |  |  |  |  | 50 |  |  |  | 50 |  |  |  |  |  |  |  |  |  | 100 |
| 15/2 | - |  |  |  |  |  | 59 |  |  |  |  | 41 |  |  |  |  |  |  |  |  |  |  |  |  | 100 |
|  | + |  |  |  |  | 43 |  |  |  |  |  | 57 |  |  |  |  |  |  |  |  |  |  |  |  | 100 |
| 16 | - |  |  |  |  |  | 62 |  |  |  |  |  |  |  |  |  |  |  |  |  |  |  |  |  | 62 |
|  | + |  |  |  |  | 51 |  |  |  |  |  | 49 |  |  |  |  |  |  |  |  |  |  |  |  | 100 |
| 16/1 | - |  |  |  |  |  |  |  |  |  |  |  |  |  |  |  |  |  |  |  |  |  |  |  | 0 |
|  | + |  |  |  |  |  |  |  |  |  |  | 100 |  |  |  |  |  |  |  |  |  |  |  |  | 100 |
| 16/2 | - |  |  |  |  |  | 50 |  |  |  |  |  |  |  |  |  |  |  |  |  |  |  |  |  | 50 |
|  | + |  |  |  |  |  |  |  |  |  |  |  |  |  |  |  |  |  |  |  |  |  |  |  | 0 |

Serial numbers of samples correspond to the numbering of locations shown in Fig. 1.
