# Supplementary material for: Overwintering of Vineyard Yeasts: Survival of Interacting Yeast Communities in Grapes Mummified on Vines
Source: Front Microbiol. 2016 Feb 29;7:212. doi: 10.3389/fmicb.2016.00212 (PMC4770031; doi:10.3389/fmicb.2016.00212)
Supplement: Supplementary file 2 [file Table2.DOC]

**TABLE S2 | The occurrence and relative abundance of basidiomyceteous species in the grape samples**

| Sample | Enrichment | % among isolates | | | | | | | | | | | | | | |
| --- | --- | --- | --- | --- | --- | --- | --- | --- | --- | --- | --- | --- | --- | --- | --- | --- |
|  |  | Agaricomycotina | | | | | | | | | Pucciniomycotina | | | |  | |
|  |  | *B. albus* | *Cr. carnescens* | *Cr. flavescens* | *Cr. magnus* | *Cr. stepposus* | *Cr. victoria* | *Cr. wieringae* | *Cryptococcus sp.* | *Ha. festucosa* | *Cu. cygneicollum* | *Cu. pallidicorallinum* | *R. graminis* | *Sp. coprosmae/oryzicola* | | Total |
| 1 | - |  |  |  |  |  |  |  |  |  |  |  |  |  | | 0 |
|  | + |  |  |  |  |  |  |  |  |  |  |  |  |  | | 0 |
| 1/1 | - |  |  |  |  |  |  |  |  |  |  |  |  |  | | 0 |
|  | + |  |  |  |  |  |  |  |  |  |  |  |  |  | | 0 |
| 1/2 | - |  |  |  |  |  |  |  |  |  |  |  | 4 | 5 | | 9 |
|  | + |  |  |  |  |  |  |  |  |  |  |  | 100 |  | | 100 |
| 2 | - |  |  |  |  |  |  |  |  |  |  |  |  |  | | 0 |
|  | + |  |  |  |  |  |  |  |  |  |  |  |  |  | | 0 |
| 2/1 | - |  |  |  |  |  |  |  |  |  |  |  |  |  | | 0 |
|  | + |  |  |  |  |  |  |  |  |  |  |  |  |  | | 0 |
| 2/2 | - |  |  |  |  |  |  |  |  |  |  |  |  |  | | 0 |
|  | + |  |  |  |  |  |  |  |  |  |  |  |  |  | | 0 |
| 3 | - |  |  |  |  |  | 22 |  |  |  |  |  |  |  | | 22 |
|  | + |  |  |  |  |  |  |  |  |  |  |  |  |  | | 0 |
| 3/1 | - |  |  |  |  |  |  |  |  |  |  |  |  |  | | 0 |
|  | + |  |  |  |  |  |  |  |  |  |  |  |  |  | | 0 |
| 3/2 | - |  |  |  |  |  |  |  |  |  |  |  |  |  | | 0 |
|  | + |  |  |  |  |  |  |  |  |  |  |  |  |  | | 0 |
| 4 | - |  |  |  |  |  |  |  |  |  |  |  |  |  | | 0 |
|  | + |  |  |  |  |  |  |  |  |  |  |  |  |  | | 0 |
| 4/1 | - |  |  | 6 |  |  |  |  |  |  |  |  |  | 26 | | 32 |
|  | + |  |  |  |  |  |  |  |  |  |  |  |  |  | | 0 |
| 4/2 | - |  |  |  |  |  |  |  |  |  |  |  |  |  | | 0 |
|  | + |  |  |  |  |  |  |  |  |  |  |  |  |  | | 0 |
| 5 | - |  |  | 34 |  |  |  |  |  |  |  |  | 10 | 10 | | 54 |
|  | + |  |  |  |  |  |  |  |  |  |  |  |  |  | | 0 |
| 5/1 | - |  |  |  |  |  | 17 |  |  |  |  | 8 |  |  | | 25 |
|  | + |  |  |  |  |  |  |  |  |  |  |  |  |  | | 0 |
| 5/2 | - |  |  | 1 |  |  |  |  |  |  |  |  |  | 4 | |  |
|  | + |  |  |  |  |  |  |  |  |  |  |  |  |  | | 0 |
| 6 | - |  |  |  | 35 |  |  |  | 50 |  |  | 15 |  |  | | 100 |
|  | + |  |  |  |  |  |  |  |  |  |  |  |  |  | | 0 |
| 6/1 | - |  |  |  |  |  |  |  |  |  |  |  |  |  | | 0 |
|  | + |  |  |  |  |  |  |  |  |  |  |  |  |  | | 0 |
| 6/2 | - |  | 38 |  | 31 |  |  |  |  |  |  |  |  | 19 | | 88 |
|  | + |  |  |  |  |  |  |  |  |  |  |  |  |  | | 0 |
| 7 | - |  |  |  |  |  |  |  |  |  |  | 17 | 1 |  | | 18 |
|  | + |  |  |  |  |  |  |  |  |  |  |  |  |  | | 0 |
| 7/1 | - |  |  |  | 9 |  |  |  |  | 11 |  | 75 |  |  | | 96 |
|  | + |  |  |  |  |  |  |  |  |  |  |  |  |  | | 0 |
| 7/2 | - |  |  | 7 |  |  |  |  |  |  |  | 9 |  |  | | 16 |
|  | + |  |  |  |  |  |  |  |  |  |  |  |  |  | | 0 |
| 8 | - |  |  |  |  |  |  |  |  |  |  |  |  |  | | 0 |
|  | + |  |  |  |  |  |  |  |  |  |  |  |  |  | | 0 |
| 8/1 | - |  |  |  |  |  |  |  |  |  | 9 | 9 |  |  | | 18 |
|  | + |  |  |  |  |  |  |  |  |  |  |  |  |  | | 0 |
| 8/2 | - |  |  |  |  |  |  |  |  |  |  |  | 3 |  | | 3 |
|  | + |  |  |  |  |  |  |  |  |  |  |  |  |  | | 0 |
| 9 | - |  |  |  |  |  |  |  |  |  |  | 59 | 22 |  | | 81 |
|  | + |  |  |  |  |  |  |  |  |  |  |  |  |  | | 0 |
| 9/1 | - |  |  |  |  |  | 32 |  |  |  |  |  |  |  | | 32 |
|  | + |  |  |  |  |  |  |  |  |  |  |  |  |  | | 0 |
| 9/2 | - |  |  |  |  |  |  |  |  |  |  |  |  |  | | 0 |
|  | + |  |  |  |  |  |  |  |  |  |  |  |  |  | | 0 |
| 10 | - |  |  |  |  |  |  |  |  |  | 71 |  |  |  | | 71 |
|  | + |  |  |  |  |  |  |  |  |  |  |  |  |  | | 0 |
| 10/1 | - |  |  |  |  |  |  |  |  |  |  |  |  |  | | 0 |
|  | + |  |  |  |  |  |  |  |  |  |  |  |  |  | | 0 |
| 10/2 | - |  |  | 50 |  | 14 |  |  |  |  | 36 |  |  |  | | 100 |
|  | + |  |  |  |  |  |  |  |  |  |  |  |  |  | | 0 |
| 11 | - |  |  |  |  |  |  |  |  |  |  | 14 |  |  | | 14 |
|  | + |  |  |  |  |  |  |  |  |  |  |  |  |  | | 0 |
| 11/1 | - |  |  |  |  |  |  |  |  |  |  | 3 |  |  | | 97 |
|  | + |  |  |  |  |  |  |  |  |  |  |  |  |  | | 0 |
| 11/2 | - |  |  |  |  |  | 13 |  |  |  |  | 14 |  |  | | 27 |
|  | + |  |  |  |  |  |  |  |  |  |  |  |  |  | | 0 |
| 12 | - |  |  |  |  |  | 24 |  |  |  |  | 34 |  |  | | 58 |
|  | + |  |  |  |  |  |  |  |  |  |  |  |  |  | | 0 |
| 12/1 | - |  |  |  |  |  |  |  |  |  |  | 57 |  | 17 | | 74 |
|  | + |  |  |  |  |  |  |  |  |  |  |  |  |  | | 0 |
| 12/2 | - |  |  | 7 |  |  | 5 |  |  |  | 7 |  |  |  | | 19 |
|  | + |  |  |  |  |  |  |  |  |  |  |  |  |  | | 0 |
| 13 | - |  |  |  |  |  | 7 |  |  |  |  | 53 |  |  | | 60 |
|  | + |  |  |  |  |  |  |  |  |  |  |  |  |  | | 0 |
| 13/1 | - | 8 |  | 5 |  |  |  | 5 |  |  |  | 62 | 8 |  | | 88 |
|  | + |  |  |  |  |  |  |  |  |  |  | 25 |  |  | | 25 |
| 13/2 | - | 2 |  |  |  |  | 9 |  |  |  |  | 15 |  |  | | 26 |
|  | + |  |  |  |  |  |  |  |  |  |  |  |  |  | | 0 |
| 14 | - |  |  |  |  |  |  |  |  |  |  | 25 |  |  | | 25 |
|  | + |  |  |  |  |  |  |  |  |  |  |  |  |  | | 0 |
| 14/1 | - |  |  | 20 |  |  |  |  |  |  | 32 |  |  |  | | 52 |
|  | + |  |  |  |  |  |  |  |  |  |  |  |  |  | | 0 |
| 14/2 | - |  |  |  |  |  |  |  |  |  |  |  |  |  | | 0 |
|  | + |  |  |  |  |  |  |  |  |  |  |  |  |  | | 0 |
| 15 | - |  |  | 28 |  |  |  |  |  |  | 36 |  |  |  | | 64 |
|  | + |  |  |  |  |  |  |  |  |  |  |  |  |  | | 0 |
| 15/1 | - |  |  | 6 |  |  |  |  |  |  |  |  |  |  | | 6 |
|  | + |  |  |  |  |  |  |  |  |  |  |  |  |  | | 0 |
| 15/2 | - |  |  |  |  |  |  |  |  |  |  |  |  |  | | 0 |
|  | + |  |  |  |  |  |  |  |  |  |  |  |  |  | | 0 |
| 16 | - |  |  |  |  |  |  |  |  |  |  | 22 | 12 | 5 | | 39 |
|  | + |  |  |  |  |  |  |  |  |  |  |  |  |  | | 0 |
| 16/1 | - |  |  |  | 38 |  |  |  |  |  |  | 48 | 14 |  | | 100 |
|  | + |  |  |  |  |  |  |  |  |  |  |  |  |  | | 0 |
| 16/2 | - |  |  |  |  |  |  |  |  |  |  |  | 50 |  | | 50 |
|  | + |  |  |  |  |  |  |  |  |  |  |  |  |  | | 0 |

Serial numbers of samples correspond to the numbering of locations shown in Fig. 1.
